# Evaluating the impact of community health volunteer home visits on child diarrhea and fever in the Volta Region, Ghana: A cluster-randomized controlled trial

**DOI:** 10.1371/journal.pmed.1002830

**Published:** 2019-06-14

**Authors:** Yeunji Ma, Christopher R. Sudfeld, Heunghee Kim, Jaeeun Lee, Yinseo Cho, John Koku Awoonor-Williams, Joseph Kwami Degley, Seungman Cha

**Affiliations:** 1 Independent Consultant, Seoul, Republic of Korea; 2 Global Health and Population Department, Harvard T.H. Chan School of Public Health, Boston, Massachusetts, United States of America; 3 Korea International Development Institute, Seoul, Republic of Korea; 4 Korea International Cooperation Agency, Seongnam-si, Republic of Korea; 5 Ghana Health Service, Accra, Ghana; 6 Department of Disease Control, Faculty of Infectious and Tropical Disease, London School of Hygiene & Tropical Medicine, London, United Kingdom; Johns Hopkins Bloomberg School of Public Health, UNITED STATES

## Abstract

**Background:**

Although there is mounting evidence demonstrating beneficial effects of community health workers (CHWs), few studies have examined the impact of CHW programs focused on preventing infectious diseases in children through behavior changes. We assessed the preventive effects of community health volunteers (CHVs), who receive no financial incentive, on child diarrhea and fever prevalence in Ghana.

**Methods and findings:**

We conducted a cluster-randomized controlled trial in 40 communities in the Volta Region, Ghana. Twenty communities were randomly allocated to the intervention arm, and 20 to the control arm, using a computer-generated block randomization list. In the intervention arm, CHVs were deployed in their own community with the key task of conducting home visits for health education and community mobilization. The primary outcomes of the trial were diarrhea and fever prevalence at 6 and 12 months among under-5 children based on caregivers’ recall. Secondary outcomes included oral rehydration treatment and rapid diagnostic testing for malaria among under-5 children, and family planning practices of caregivers. Generalized estimating equations (GEEs) with a log link and exchangeable correlation matrix were used to determine the relative risk (RR) and 95% confidence intervals (CIs) for diarrhea, fever, and secondary outcomes adjusted for clustering and stratification. Between April 18 and May 4, 2015, 1,956 children were recruited and followed up until September 20, 2016. At 6 and 12 months post-randomization, 1,660 (85%) and 1,609 (82%) participants, respectively, had outcomes assessed. CHVs’ home visits had no statistically significant effect on diarrhea or fever prevalence at either time point. After a follow-up of 12 months, the prevalence of diarrhea and fever was 7.0% (55/784) and 18.4% (144/784), respectively, in the control communities and 4.5% (37/825) and 14.7% (121/825), respectively, in the intervention communities (12-month RR adjusted for clustering and stratification: diarrhea, RR 0.73, 95% CI 0.37–1.45, *p =* 0.37; fever, RR 0.76, 95% CI 0.51–1.14, *p =* 0.20). However, the following were observed: improved hand hygiene practices, increased utilization of insecticide-treated bed nets, and greater participation in community outreach programs (*p*-values < 0.05) in the intervention group. In a post hoc subgroup analysis, the prevalence of diarrhea and fever at 6 months was 3.2% (2/62) and 17.7% (11/62), respectively, in the intervention communities with ≥70% coverage and a ≥30-minute visit duration, and 14.4% (116/806) and 30.2% (243/806) in the control communities (RR adjusted for clustering, stratification, baseline prevalence, and covariates: diarrhea, RR 0.23, 95% CI 0.09–0.60, *p =* 0.003; fever, RR 0.69, 95% CI 0.52–0.92, *p =* 0.01). The main limitations were the following: We were unable to investigate the longer-term effects of CHVs; the trial may have been underpowered to detect small to moderate effects due to the large decline in diarrheal and fever prevalence in both the intervention and control group; and caregivers’ practices were based on self-report, and the possibility of caregivers providing socially desirable responses cannot be excluded.

**Conclusions:**

We found no effect of CHVs’ home visits on the prevalence of child diarrhea or fever. However, CHV programs with high community coverage and regular household contacts of effective duration may reduce childhood infectious disease prevalence.

**Trial registration:**

International Standard Randomised Controlled Trial Registry, ISRCTN49236178.

## Author summary

**Why was this study done?:**

- A wide range of health interventions could be delivered by community health workers (CHWs) including interventions for nutrition, maternal health, birth and newborn care preparedness, neonatal health, and promotion of breastfeeding. Many of the previous studies of CHW interventions have focused on case management, immunization, or child mortality.
- Only a few randomized controlled trials have explored the promotive effect of CHWs on infectious disease in children through health education, particularly when CHWs were assigned comprehensive tasks on a voluntary basis, including community mobilization, home visits, minor illness treatment, support of the professional health workforce, surveillance, and referral of patients to health facilities, and the results were mixed.

**What did the researchers do and find?:**

- We conducted a cluster-randomized controlled trial in 40 communities in Ghana to assess the effect of a community health volunteer (CHV) intervention on reducing diarrhea and fever prevalence in children under 5 years of age, and the participants were followed up at 6 and 12 months after the intervention started. Associations of CHVs’ home visit coverage and intensity with the primary outcomes, 14-day diarrhea and fever prevalence, were also examined.
- No statistically significant effect of CHVs on the primary outcomes was found; however, we found significant differences in some health behaviors to prevent diarrhea and malaria between the intervention and control groups. We found that the communities in which the CHV intervention was fully implemented had lower risks of both diarrhea and fever compared to control communities.

**What do these findings mean?:**

- We found that CHVs’ home visits had no overall effect on prevention and treatment of child diarrhea and fever, or on family planning practices.
- However, a subgroup analysis identified potential beneficial effects among villages where the CHV intervention reached ≥70% coverage of home visits, with an average visit duration of ≥30 minutes.
- This study has policy implications for CHW home visit programs, particularly regarding the frequency and duration of home visits and the optimal number of educational messages to be delivered.

## Introduction

Globally, 5.4 million deaths occurred among children younger than 5 years in 2017; diarrhea and malaria are estimated to have caused 533,800 and 266,000 of these deaths, respectively [1,2]. Although a substantial number of child deaths from diarrhea and malaria could be averted by existing interventions, many low- and middle-income countries have suboptimal coverage of these interventions and face a severe shortage of the workforce needed to deliver essential health services [3]. Accordingly, the global health community has renewed its interest in the potential contributions of community health workers (CHWs) [4].

A wide range of interventions can be delivered by CHWs, including nutritional, neonatal health, and maternal health interventions [5]. However, although there is mounting evidence demonstrating the beneficial effect of CHWs on disease treatment, breastfeeding, and overall child mortality, few studies have examined the impact of CHW programs focused on preventing infectious diseases in children through behavior change communication [6–8]. A recent systematic review [5] identified only 4 randomized controlled trials that were conducted to assess the effects of CHWs on childhood illnesses in low- and middle-income countries in which the primary outcome was immunization coverage or case management, and found mixed results [9–11]. A trial [10] in Malawi found that volunteer counseling through home visits led to a significant reduction in reported infant cough, fever, or diarrhea, whereas there was no effect of a community-based worker program on child cough, fever, or diarrhea prevalence in a trial [9] conducted in India. Overall, the effectiveness of CHWs for preventing routine childhood diseases remains unclear.

In 2005, Ghana adopted the Community-based Health Planning and Services (CHPS) initiative, which intends to improve the health of people living in rural areas of the country [12]. Two cadres of CHWs collaborate to provide primary healthcare in the CHPS system in Ghana: community health nurses (CHNs) and community health volunteers (CHVs). CHNs are trained for 2 years as licensed nurses and recruited as salaried government employees, while CHVs are trained for less than 6 weeks and do not receive any financial incentives. CHVs are tasked with providing health education, birth and death registration, disease surveillance, and minor illness treatment. The effects of the Ghana CHV program on child health remain unknown [13]. In order to address this gap, we present a cluster-randomized trial that examined the effect of CHVs on child diarrhea and fever prevalence in rural Ghana. We also explore whether community coverage and duration of home visits influence the impact of the CHV program.

## Methods

### Study design and participants

We conducted a cluster-randomized controlled trial in 40 communities (villages) of the Ketu South District in the Volta Region, Ghana, between February 1, 2015 and September 20, 2016 (ISRCTN49236178) [14]. The baseline survey, and recruitment of caregivers (mother or primary female caregiver) and under-5 children, began on April 18, 2015, and the trial was registered on June 16, 2015. Randomization was performed in June 2015 using the baseline survey results, and the intervention started on August 15, 2015. The estimated population of the Ketu South District in 2015 was 181,881, and the number of children aged under 5 years was 36,376. Communities in the study area had a range of 130–254 households. A phase-in design was adopted for the trial, wherein the CHVs were recruited and activated in the 20 intervention communities during the trial period, while the 20 communities in the control arm received CHVs after completion of the end line survey of the trial. Ethical approval for the trial was obtained from the Ghana Health Service Ethics Review Committee (GHS-ERC:07/01/15), and the evaluation was supplementarily approved by the Harvard T.H. Chan School of Public Health (IRB17-2051).

We assured allocation concealment for participants by selecting them before randomizing clusters [15]. The study recruited participants from April 18 to May 4, 2015. The inclusion criteria for the trial were households located in the trial catchment area that had at least 1 child under 5 years of age. The best estimate of each child’s age was determined by health card, insurance card, or caregiver’s report. All caregivers of the participants were informed of their right to withdraw from the study at any time. Children of caregivers who declined to participate in the surveys were excluded from the study. Study participants were enrolled for 16 months, and outcome data collection occurred at 6 and 12 months after study initiation. In households with more than 1 child, we recruited the youngest child. Children were not censored at the following surveys if they became greater than 60 months of age.

### Randomization

A cluster randomization was chosen to prevent contamination in this study. The community level, where people interact with one another most closely, was taken as the randomization unit. To minimize the possibility of selection bias, we identified and recruited clusters before randomization [16]. Rural communities in the Ketu South District were identified for potential participation in the trial. Among the 57 rural communities that were identified, 40 were randomly selected using probability-proportionate-to-size methods to be included in the trial. In the second stage, stratified randomization was used, based on the baseline survey results, to assign communities to either intervention or control and reduce the risk of baseline imbalances. The 40 selected communities were stratified into 8 strata based on the estimated diarrheal prevalence among under-5 children, the economic status of the community, and the proportion of caregivers who had skilled delivery for their youngest child. To estimate the household economic status of a community, the proportion of housing structures with wattle and daub construction was used as a proxy indicator representing low-economic-status communities (cutoff point: 55.0%). Within the 8 strata, 20 communities were randomly allocated to the intervention arm, and 20 to the control arm, using a computer-generated block randomization list (by YM and SC).

### Sample size

On the basis of a preliminary survey undertaken in 2013, the prevalence of both child diarrhea and malaria was estimated to be 25%, and was assumed to be reduced by 25% by the intervention based on a previous study [6,9,10]. We estimated the coefficient of variation to be 0.16. With a 10% attrition rate, the required sample size was 950 households across 20 clusters per arm, with a study power of 80% [17].

### Intervention

Details of the CHV intervention and characteristics of the CHVs are summarized in S1 Text. We incorporated multiple activities into the program that have been recommended to promote CHVs’ effectiveness (e.g., training and retraining, selecting CHVs among those most respected by the community, household education with visual aids, material rewards as incentives, regular supervision by health professionals, collaboration with the existing health system, and community awareness of CHVs) [3,18].

All CHVs’ activities were delivered within the community setting. The CHVs’ core task was to carry out home visits to each household every 2 months. They were recommended to spend at least 30 minutes on each household visit. While visiting households, CHVs were instructed to provide health education based on 10 key messages using visual aids. The messages covered actionable recommendations to improve the household members’ knowledge and behavior related to maternal and child health. They included information on the prevention of diarrhea and malaria, more specifically, that proper handwashing and improved sanitation and hygiene can prevent diarrhea, and sleeping under insecticide-treated bed nets (ITNs) can prevent malaria. CHVs also provided messages about how to manage diarrhea and malaria in children, more specifically, that children with diarrhea should be given oral rehydration salts (ORS) and taken to a health facility and that suspected malaria cases should be properly diagnosed and treated at a health facility. The key messages also included the benefits of using contraceptive methods and information on types of contraception, as well as the importance of participating in the community outreach program (child welfare clinic). Another major task of CHVs during home visits was to give ORS to children with diarrhea and to perform a malaria test if any child had a fever. CHVs were also recommended to support CHNs and to mobilize community members for child welfare clinics that were held monthly in the community.

After the communities were randomly allocated to the intervention and control arms, CHVs were recruited from the intervention communities. The CHV selection process was coordinated by the Ketu South District Health Management Team (DHMT) in cooperation with community committees. The committees were requested to nominate candidates based on their literacy level, volunteerism, and experience. Regional and district health officials trained the CHVs for 5 days, including 2 days of field-based training to practice home visit skills and outreach support. The content of the training curriculum was prepared through discussions among the regional and district health teams and the project team. The objective of the CHVs’ training was to help them to understand the concept of CHPS, their roles and responsibilities, and essential maternal and child health services. The training also aimed to teach skills to properly conduct home visits and provide health education using 10 key messages, malaria tests using a rapid diagnostic test (RDT), ORS, and referral of patients to their supervisor. Refresher training was undertaken every month by the DHMT members and CHNs [14]. After the training, community leaders, together with DHMT officials, held community meetings to introduce the CHVs and to publicly declare the CHVs’ roles and position. The CHVs, who were the implementers of the program, were mainly community-based men and women who had graduated from junior high school, and they were rewarded with material compensation in the form of cell phone minutes and food items. CHNs treated patients who visited the CHPS compound and undertook community outreach programs to provide vaccinations, nutrition, and health education activities on a monthly basis. Community people and the project team were not blinded to the intervention because of the distinctive nature of CHV home visits.

### Data collection

Three rounds of household surveys were conducted to evaluate the effect of the CHV program. The data collection team consisted of 14 data collectors and 2 supervisors, and all were blinded to whether a community was randomized to the intervention or control arm. All data collectors were trained for 2–3 days before every round of the survey, and participated in daily review sessions during the data collection period for quality assurance. The baseline survey was conducted from April 18 to May 4, 2015, before the start of the CHV intervention on August 15, 2015. The first follow-up survey was administered from February 6, 2016, after 6 months of intervention, and the end line survey was conducted from September 5, 2016, after 12 months of intervention.

A set of questionnaires was developed and used to conduct the survey. The survey instrument primarily consisted of questions assessing the socioeconomic status of households and the prevalence of children’s diarrhea and febrile illness based on caregivers’ reports. Another key element was questions on CHVs’ activities reported by caregivers, including the frequency and duration of their home visits. The survey also contained a series of questions that gathered information about caregivers’ experiences of family planning, such as the use of various types of contraceptive methods, as well as information about caregivers’ most recent delivery, such as the place of delivery and utilization of antenatal and postnatal care.

We applied systematic sampling to approach households in the communities. The data collectors visited households in each community using the interval method, in which the total number of households in a community is divided by the cluster size. They started by visiting a household located nearest to the main road, from which they continued to visit the next *n*th household based on the interval (e.g., the next fifth household if the interval was 5). Data collectors asked any household members if they had at least 1 under-5 child. If the household did not meet the eligibility criterion of having at least 1 child under 5 years old, the data collectors visited the next house and continued visiting the next *n*th one. At each visited household, the primary female caregiver of the youngest child in the household was asked to participate in the survey, and signed the consent form for enrollment if she agreed.

### Process evaluation

Among the 1,956 households enrolled for impact evaluation, 408 (21%) were randomly selected and registered for process evaluation after obtaining separate informed consent for this additional survey. The components of the process evaluation were developed based on the framework of Steckler and Linnan [19]. Data on the indicators of each step of the intervention process were collected through a combination of methods, including household surveys, documentation review, and direct observation. Four rounds of household surveys were carried out at 3-month intervals. For the sample size, we referred to previous studies [20,21] of process evaluation in randomized controlled trials.

A combination of self-reporting and direct observation was used to assess process indicators. Direct observation accompanied the household surveys for health behaviors such as appropriate use of ITNs and proper handwashing. Those who responded that they had used ITNs for their child during the previous night at the time of the survey were only considered to have actually done so if an ITN was hung up inside their house. For diarrhea, after administering standard questions about caregivers’ handwashing practices in the previous 24 hours at the time of the survey, direct observations were made. Only when caregivers were observed to wash their hands with running water and soap, upon request of demonstration, were they considered to practice appropriate hand hygiene.

Caregivers’ participation in community outreach program (child welfare clinic) and child growth checks was surveyed. Among the 10 key messages delivered by the CHVs through home visits, we examined which specific messages were recalled by caregivers. In addition, various process indicators of intervention fidelity were assessed, including the results of 4 rounds of CHVs’ tests, the CHV monthly review meeting attendance rate, the proportion of CHVs with regular recording of logbooks, CHVs’ self-reported coverage of home visits, CHVs’ participation in community-wide health activities, and the rate of CHVs’ retention in their duties at 3, 6, 9, and 12 months. For these indicators, CHVs’ logbooks, monthly review meeting minutes, CHN outreach records, referral records, and the CHPS inventory logs were investigated.

### Statistical analysis

The primary outcomes of the trial were caregiver report of 14-day diarrhea and fever prevalence among under-5 children. Diarrhea was defined as having 3 or more instances of watery stools within 24 hours in the past 14 days, and we used febrile episodes in the last 14 days as a proxy indicator of malaria prevalence. Family planning practices and case management for child diarrhea and fever were investigated as secondary outcomes. For family planning practices, we examined long-term and short-term contraceptive methods (i.e., female/male sterilization, intrauterine devices, injectable contraceptives, implants, pills, female/male condoms, the standard-days method, the rhythm method, the lactational amenorrhea method, and withdrawal). For child diarrhea, we investigated whether the sick child was administered ORS, and for fever, we investigated whether the child was tested for malaria with a RDT kit. When designing the trial, we planned to examine the effect of the intervention on antenatal and postnatal care, as well as on case management of malaria of pregnant women as secondary outcomes. However, effects on these outcomes were not analyzed because they were extremely underpowered due to the small number of pregnancies in the registered households during the intervention (S3 Table).

Baseline caregiver, child, and household characteristics were assessed for comparability between randomized arms. The statistical analyses for the primary and secondary endpoints were based on the intention-to-treat analysis principle. Generalized estimating equations (GEEs) with a log link and exchangeable correlation matrix were used to assess the relative risk (RR) and 95% confidence intervals (CIs) for diarrhea, fever, and secondary outcomes adjusted for clustering and stratification. Fixed-effect covariates were used to account for variables used in the stratified randomization design. We also conducted sensitivity analyses controlling for potential baseline imbalances in factors such as baseline diarrhea or fever prevalence, household income quintile, caregivers’ education, child age and sex, access to an improved water source, and improved sanitation. If the RR of the baseline-covariate-adjusted model differed from that of the unadjusted model by greater than 10%, we presented the adjusted model as the primary model.

In addition, we examined associations of CHVs’ home visit coverage and intensity with primary outcomes. We presented observational relationships stratified by coverage (whether 70% or more of households received CHV home visits at least once in the past 3 months) and duration of visit (whether the average time spent on each home visit in a community was ≥30 minutes), as well as the combination of these factors. For the coverage and duration of household visits, we used caregivers’ reports gathered from household surveys. Due to the observational nature of these analyses, the analyses were adjusted for baseline fever or diarrhea, income quintile, caregiver education, child sex, and child age. All statistical analyses were conducted using STATA version 13. *p*-Values < 0.05 were considered to indicate statistical significance.

## Results

### Baseline characteristics of participants

Between April and May 2015, 1,970 caregivers of children under 5 years were approached for participation in the trial, of whom 1,956 (99.3%) consented and participated in the trial. The baseline characteristics of the trial participants, caregivers, and households are presented in Table 1. The intervention and control groups appeared to be comparable at baseline. Around 23% of caregivers had completed primary education, and more than 50% had not, in both arms. The mean age of the registered children was 23 months (SD 16), and 51% were male. Approximately 64% of households had an improved water source (sachet water, borehole, bottled water, or public standpipe), and roughly 24% of households had access to improved sanitation (Kumasi Ventilated Improved Pit latrine or flush toilet).

**Table 1:** Baseline characteristics of community health volunteer intervention (*n =* 20 clusters; 999 children; mean cluster size 49 children) and control clusters (*n =* 20 clusters; 957 children; mean cluster size 47 children).

Characteristic	Intervention		Control	
	*N*	Percent or mean (SD)	*N*	Percent or mean (SD)
Child age (months)	989	23.0 (16.0)	946	22.0 (15.9)
Child sex: male	505	50.6%	483	50.5%
Mother or primary caregiver age (years)	999	29.0 (7.2)	957	28.9 (6.8)
Education level of caregiver				
None/did not complete primary	455	57.2%	406	55.3%
Completed primary school	188	23.6%	165	22.5%
Completed secondary school	19	2.4%	24	3.3%
Possession of National Health Insurance Scheme card	323	33.2%	328	34.3%
Possession of child health record book	689	69.0%	671	70.1%
Main source of drinking water				
Sachet water	202	20.2%	223	23.3%
Borehole	190	19.0%	141	14.7%
Bottled water	163	16.3%	150	15.7%
Public standpipe	70	7.0%	96	10.0%
Unimproved sources	364	36.8%	336	35.5%
Household sanitation				
KVIP*	213	21.5%	188	19.9%
Flush toilet	38	3.8%	26	2.7%
Unimproved sources	738	74.6%	732	77.4%

*Kumasi Ventilated Improved Pit latrine.

### Trial profile

**Fig 1:**
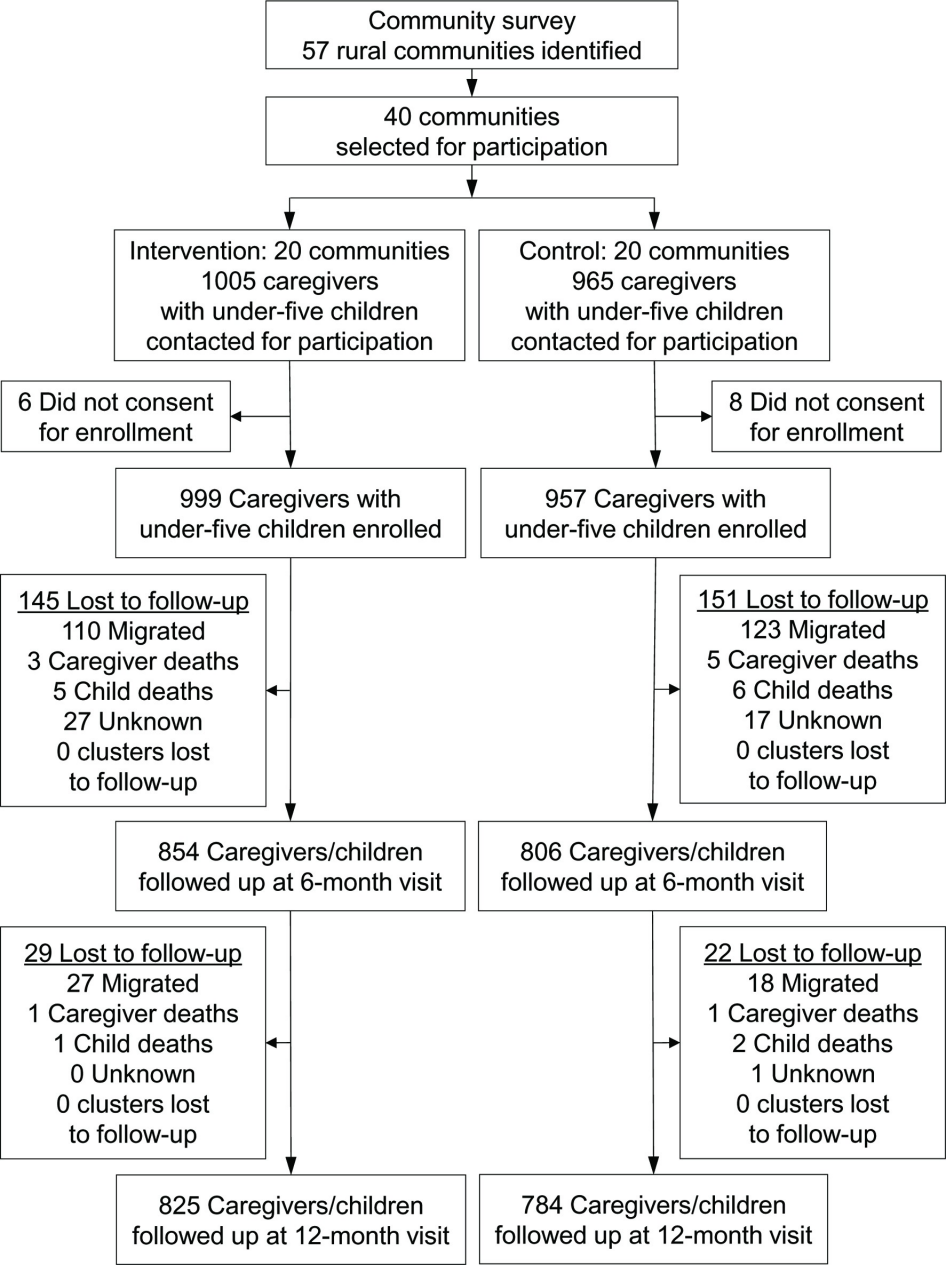
Trial profile.

We followed up participants at 6 and 12 months post-randomization. At 6 months, 1,660 (85%) had outcomes assessed, and 1,609 (82%) had outcomes assessed at 12 months. The attrition rate at 12 months was similar between the intervention (17.4%) and control (18.1%) arms. Among the 347 randomized participants without outcome data at 12 months, 278 (80.1%) had relocated or seasonally migrated out of the study area, 10 (2.9%) caregivers and 14 (4.0%) children had died, and 45 (13.0%) had unknown status (Fig 1). Among the 77 CHVs who were initially recruited, 87% were retained until 12 months; the demographic and socioeconomic profiles of the CHVs are presented in S1 Text. We found no difference in the characteristics of the caregivers and children who were retained in the trial at 6 months and 12 months and those who were lost to follow-up (e.g., caregivers’ age or education level, income quintile, child’s age or sex, water source, and sanitation status).

### Effects of CHVs on the primary and secondary outcomes

Table 2 presents the effects of CHVs on the primary outcomes of 14-day prevalence of diarrhea and fever at 6 and 12 months of follow-up. There was no statistically significant effect of CHVs on 14-day diarrhea or fever prevalence at either time point (diarrhea: 6-month RR 0.79, 95% CI 0.53–1.15, *p =* 0.21; 12-month RR 0.73, 95% CI 0.37–1.45, *p =* 0.37; fever: 6-month RR 0.95, 95% CI 0.68–1.33, *p =* 0.77; 12-month RR 0.76, 95% CI 0.51–1.14, *p =* 0.20). We also found no overall effect on the secondary outcomes of family planning, ORS treatment for diarrhea cases, or malaria testing for fever cases. These findings were robust in the adjusted analysis, which accounted for potential baseline imbalances between intervention and control villages (Table 2). In sensitivity analyses, we also found no effect on 14-day diarrhea (RR 0.77, 95% CI 0.51–1.16, *p =* 0.21) or fever (RR 0.87, 95% CI 0.64–1.18, *p =* 0.38) prevalence when using a longitudinal GEE analysis that included both time points (6 and 12 months of follow-up) in the same model (S1 Table). The coefficient of variation of diarrhea at baseline was 0.60.

**Table 2:** Effect of the community health volunteer program on primary outcomes of diarrhea and fever and secondary outcomes of malaria testing for fever, oral rehydration salts (ORS) treatment for diarrhea, and family planning practices, at 6 and 12 months of follow-up.

Outcome	Baseline		6-month follow-up						12-month follow-up					
	Intervention (*N =* 999) *n/N* (%)	Control (*N =* 957) *n/N* (%)	Intervention (*N =* 854) *n/N* (%)	Control (*N =* 806) *n/N* (%)	Relative risk* (95% CI)	*p*-Value	Adjusted relative risk^†^ (95% CI)	*p*-Value	Intervention (*N =* 825) *n/N* (%)	Control (*N =* 784) *n/N* (%)	Relative risk* (95% CI)	*p*-Value	Adjusted relative risk^†^ (95% CI)	*p*-Value
***Primary outcomes***														
14-day diarrhea prevalence	175/999 (17.5%)	192/957 (20.1%)	96/854 (11.2%)	116/806 (14.4%)	0.79 (0.53–1.15)	0.21	0.79 (0.55–1.14)	0.21	37/825 (4.5%)	55/784 (7.0%)	0.73 (0.37–1.45)	0.37	0.74 (0.38–1.46)	0.39
14-day fever prevalence	294/999 (29.4%)	337/957 (35.2%)	223/854 (26.1%)	243/806 (30.2%)	0.95 (0.68–1.33)	0.77	0.99 (0.72–1.38)	0.97	121/825 (14.7%)	144/784 (18.4%)	0.76 (0.51–1.14)	0.20	0.77 (0.53–1.12)	0.17
***Secondary outcomes***														
ORS for diarrhea cases	73/175 (41.7%)	65/192 (33.9%)	47/96 (49.0%)	62/116 (53.5%)	1.00 (0.82–1.23)	0.97	0.95 (0.79–1.13)	0.55	19/37 (51.4%)	34/55 (61.8%)	0.85 (0.56–1.31)	0.47	0.76 (0.51–1.14)	0.18
Malaria test for fever cases	75/294 (25.5%)	72/337 (21.4%)	79/223 (35.4%)	85/243 (35.0%)	1.01 (0.74–1.38)	0.95	1.18 (0.77–1.82)	0.45	49/121 (40.5%)	60/144 (41.7%)	1.08 (0.75–1.56)	0.68	1.07 (0.65–1.78)	0.79
Family planning^‡^	313/904 (34.6%)	288/865 (33.3%)	303/794 (38.2%)	300/747 (40.2%)	0.93 (0.80–1.09)	0.38	0.95 (0.83–1.09)	0.49	332/776 (42.8%)	282/711 (39.7%)	1.02 (0.86–1.21)	0.79	1.06 (0.93–1.22)	0.37

*Accounting for cluster and stratified randomization.

^†^Diarrhea: adjusted for baseline diarrhea, income quintile, caregiver’s education, child sex, child age, water source, sanitation status, clustering effect, and stratification; fever: adjusted for baseline fever, income quintile, caregiver’s education, child sex, child age, clustering effect, and stratification; ORS treatment: adjusted for baseline treatment, child sex, child age, caregiver’s education, income quintile, clustering effect, and stratification (age, education, and income quintile were not adjusted for the 12-month analysis due to diverging estimates); malaria test: adjusted for baseline test, child sex, child age, caregiver’s education, clustering effect, and stratification; family planning: adjusted for baseline family planning, caregiver’s age, caregiver’s education, clustering effect, and stratification.

^‡^Family planning was defined as the use of long-term or short-term contraceptive methods (i.e., female/male sterilization, intrauterine devices, injectable contraceptives, implants, pills, female/male condoms, the standard-days method, the rhythm method, the lactational amenorrhea method, or withdrawal).

### Effects of the CHV intervention on health behaviors

Table 3 presents the effect of the CHV intervention on health behaviors. We found significant differences in some health behaviors between the intervention and control groups. Caregivers in the intervention group were more likely to wash their hands with running water and soap at 5 critical times than the control group (RR 1.39, 95% CI 1.15–1.68, *p =* 0.001). In addition, CHVs had increased utilization of ITNs for under-5 children (RR 1.06, 95% CI 1.01–1.12, *p =* 0.02). The caregivers in the intervention communities also participated more frequently in community outreach programs (child welfare clinic) (RR 1.22, 95% CI 1.06–1.42, *p =* 0.007). No adverse or harmful events with regards to CHV home visits were reported in this study [22].

**Table 3:** Effect of the community health volunteer program on caregiver handwashing behavior, mosquito net utilization for children under 5 years, and community-wide health activity participation.

Health behavior	3 months		6 months		9 months		12 months		Relative risk* (95% CI)	*p*-Value	Adjusted relative risk^†^ (95% CI)	*p*-Value
	Intervention	Control	Intervention	Control	Intervention	Control	Intervention	Control				
	(*N =* 208)	(*N =* 200)	(*N =* 188)	(*N =* 183)	(*N =* 183)	(*N =* 175)	(*N =* 168)	(*N =* 168)				
***Handwashing behavior***												
Before cooking	170	150	178	176	181	172	155	152	1.02	0.30	1.02	0.20
	(81.7%)	(75.0%)	(94.7%)	(96.2%)	(98.9%)	(98.3%)	(92.3%)	(90.5%)	(0.98–1.05)		(0.99–1.06)	
Before feeding	165	143	150	139	179	155	93	101	1.05	0.10	1.04	0.17
	(79.3%)	(71.5%)	(79.8%)	(76.0%)	(97.8%)	(88.6%)	(55.4%)	(60.1%)	(0.99–1.12)		(0.98–1.11)	
After defecating	179	163	186	179	181	170	162	160	1.01	0.64	1.02	0.07
	(86.1%)	(81.5%)	(98.9%)	(97.8%)	(98.9%)	(97.1%)	(96.4%)	(95.2%)	(0.96–1.07)		(1.00–1.05)	
Before eating	182	154	176	172	180	166	149	144	1.04	0.04	1.04	0.03
	(87.5%)	(77.0%)	(93.6%)	(94.0%)	(98.4%)	(94.9%)	(88.7%)	(85.7%)	(1.00–1.08)		(1.01–1.08)	
After hand-shaking (at community activities)	80	58	33	39	94	59	41	23	1.39	<0.001	1.38	0.001
	(38.5%)	(29.0%)	(17.6%)	(21.3%)	(51.4%)	(33.7%)	(24.4%)	(13.7%)	(1.16–1.67)		(1.15–1.66)	
Handwashing at all 5 critical times	73	56	30	37	93	54	29	16	1.39	0.001	1.36	0.002
	(35.1%)	(28.0%)	(16.0%)	(20.2%)	(50.8%)	(30.9%)	(17.3%)	(9.5%)	(1.15–1.68)		(1.12–1.65)	
***Malaria prevention***												
Insecticide-treated bed net utilization for child	188	174	150	135	172	154	144	136	1.06	0.02	1.06	0.04
	(90.4%)	(87.0%)	(79.8%)	(73.8%)	(94.0%)	(88.0%)	(85.9%)	(81.0%)	(1.01–1.12)		(1.00–1.12)	
***Community participation***												
Participated in community outreach program (child welfare clinic)	124	98	77	52	99	73	63	59	1.22	0.007	1.21	0.01
	(59.6%)	(49.0%)	(41.0%)	(28.4%)	(54.1%)	(41.7%)	(37.5%)	(35.1%)	(1.06–1.42)		(1.04–1.41)	
Participated in child growth check in the last month	114	111	102	80	113	93	70	70	1.06	0.04	1.16	0.03
	(54.8%)	(55.5%)	(54.6%)	(44.2%)	(61.8%)	(53.1%)	(41.7%)	(41.7%)	(1.00–1.33)		(1.01–1.33)	

*Data source: A cohort of 336 caregivers was followed up for 1 year with 4 rounds of surveys including direct observation (18% attrition rate).

^†^Adjusted for household income quintile, caregiver’s education, and caregiver’s age (for “after defecating,” only the education level was adjusted for because estimates diverged when adjusted for income quintile, education level, and age).

### Degree of exposure to the CHVs at household and cluster levels

Table 4 shows the degree of exposure to the CHVs in the intervention group at the household and cluster levels. The percentage of households that received a CHV visit at least once in the past 3 months was similar at the 6-month and the 12-month time points—51.5% and 59.2%, respectively. The mean duration of the visits declined from 31 minutes to 18 minutes from 6 to 12 months. Only 2 communities had fully activated CHVs at 6 months, defined as 70% or more of households in the cluster reporting that a CHV visited at least once in the past 3 months and that the mean length of the visits was 30 minutes or longer. No communities had fully activated CHVs at 12 months, primarily due to the decline in visit duration. We also saw that the mean number of the 10 key program messages recalled by caregivers in the intervention group decreased from 5.3 to 3.8 messages from 6 to 12 months (Table 4). Furthermore, we saw declines in key message recall for all of the 10 messages (S2 Table). Other process indicators of intervention fidelity are described in S1 Text (i.e., the results of 4 rounds of CHVs’ tests, the CHV monthly review meeting attendance rate, the proportion of CHVs with regular recording of logbooks, CHVs’ self-reported coverage of home visits, CHVs’ participation in community-wide health activities, and the rate of CHVs’ retention in their duties at 3, 6, 9, and 12 months).

**Table 4:** Household- and cluster-level degree of exposure to CHVs based on caregivers’ recall at 6 and 12 months of follow-up.

Measure	6-month follow-up	12-month follow-up
***Household level***		
CHV visit at least once in the past 3 months	440/854 (51.5%)	488/825 (59.2%)
Mean duration of CHV visit (minutes)	31 (SD 29)	18 (SD 14)
Mean number of program messages recalled out of 10	5.3 (SD 2.6)	3.8 (SD 1.9)
***Cluster level***		
≥70% of the households in the cluster received a CHV visit at least once in the past 3 months	5/20 (25.0%)	11/20 (55.0%)
Mean CHV visit duration ≥ 30 minutes	9/20 (45.0%)	0/20 (0%)
≥70% of the households in the cluster received a CHV visit at least once in the past 3 months and mean CHV visit duration ≥ 30 minutes	2/20 (10.0%)	0/20 (0%)

CHV, community health volunteer.

### Subgroup analysis of the CHV intervention and 14-day diarrhea and fever prevalence

We conducted a subgroup analysis that examined the association of community-level coverage and CHV visit duration with the primary outcomes of 14-day diarrhea and fever prevalence (Table 5). Due to the observational nature of this analysis, we adjusted for potential confounders. Overall, we found no difference in diarrhea and fever prevalence in the intervention and control groups. However, in communities where caregivers had a mean CHV visit duration ≥ 30 minutes, the child diarrheal prevalence was significantly lower than in the control group at 6 months (RR 0.62, 95% CI 0.39–0.99, *p =* 0.04). Furthermore, communities in which the CHV intervention was fully implemented, i.e., with ≥70% coverage and a mean duration of CHV visits ≥ 30 minutes, had significantly lower risks of both diarrhea (RR 0.23, 95% CI 0.09–0.60, *p =* 0.003) and fever (RR 0.69, 95% CI 0.52–0.92, *p =* 0.01) than the control group.

**Table 5:** Analysis of CHV program effectiveness on 14-day diarrhea and fever prevalence within subgroups by within-cluster coverage and mean duration of CHV visit.

Outcome	6-month follow-up				12-month follow-up			
	Intervention	Control	Adjusted* relative risk (95% CI)	*p*-Value	Intervention	Control	Adjusted* relative risk (95% CI)	*p*-Value
***Subgroup analysis by within-cluster percent of households that received a CHV visit at least once in the past 3 months***								
Diarrhea								
≥70% of households	31/215 (14.4%)	14.4%	0.98 (0.64–1.48)	0.91	19/380 (5.0%)	7.0%	0.69 (0.31–1.55)	0.37
<70% of households	65/639 (10.2%)	14.4%	0.73 (0.48–1.12)	0.15	18/445 (4.0%)	7.0%	0.80 (0.39–1.67)	0.56
Fever								
≥70% of households	67/215 (31.2%)	30.2%	1.16 (0.80–1.67)	0.44	48/380 (12.6%)	18.4%	0.64 (0.40–1.03)	0.07
<70% of households	156/639 (24.4%)	30.2%	0.94 (0.66–1.35)	0.74	73/445 (16.4%)	18.4%	0.88 (0.60–1.30)	0.53
***Subgroup analysis by within-cluster mean CHV visit duration***								
Diarrhea								
≥30 minutes	33/402 (8.2%)	14.4%	0.62 (0.39–0.99)	0.04	0/0			
<30 minutes	63/452 (13.9%)	14.4%	0.92 (0.61–1.40)	0.71	37/825 (4.5%)	7.0%	0.74 (0.38–1.46)	0.39
Fever								
≥30 minutes	89/402 (22.1%)	30.2%	0.82 (0.58–1.14)	0.24	0/0			
<30 minutes	134/452 (29.7%)	30.2%	1.02 (0.69–1.52)	0.92	121/825 (14.7%)	18.4%	0.77 (0.53–1.12)	0.17
***Subgroup analysis by within-cluster percent of households that received a CHV visit at least once in the past 3 months and mean CHV visit duration***								
Diarrhea								
≥70% of households & ≥30 minutes	2/62 (3.2%)	14.4%	0.23 (0.09–0.60)	0.003	0/0	7.0%	—	
<70% of households or <30 minutes	94/792 (11.9%)	14.4%	0.85 (0.60–1.20)	0.35	37/825 (4.5%)	7.0%	0.74 (0.38–1.46)	0.39
Fever								
≥70% of households & ≥30 minutes	11/62 (17.7%)	30.2%	0.69 (0.52–0.92)	0.01	0/0	7.0%	—	
<70% of households or <30 minutes	212/792 (26.8%)	30.2%	0.95 (0.68–1.34)	0.78	121/825 (14.7%)	18.4%	0.77 (0.53–1.12)	0.17

*Diarrhea: adjusted for baseline diarrhea, income quintile, caregiver’s education, child sex, child age, water source, sanitation status, stratified randomization, and clustering; Fever: adjusted for baseline fever, income quintile, caregiver’s education, child sex, child age, stratified randomization, and clustering.

CHV, community health volunteer.

## Discussion

Overall, we found no effect of the CHV home visit intervention on 14-day diarrhea and fever prevalence at 6 and 12 months of follow-up. In terms of secondary outcomes, the following were observed: improved hand hygiene practices, increased utilization of ITNs, and greater participation in community outreach programs in the intervention group. There was no effect on family planning, ORS treatment for diarrhea cases, or malaria testing for fever cases. We found that the coverage of the CHV intervention was suboptimal, and the duration of CHV visits declined over time, in tandem with caregivers’ recall of key program messages. In an observational subgroup analysis, we found that communities with ≥70% coverage of the CHV intervention and average visits lasting ≥30 minutes had significantly lower diarrhea and fever prevalence than the control communities.

First, it is important to note that in our study, we found large reductions in child diarrhea and fever prevalence over time in both the intervention and control groups over the 12 months of follow-up. In the control group, the prevalence of diarrhea and fever from baseline to 12 months decreased from 20.1% to 7.0% and from 35.2% to 18.4%, respectively. It is impossible to determine the specific reasons for this decline, but we hypothesize that it was the result of the overall community health program implemented district-wide. The health program supported training of CHNs to enhance health promotion in their communities, provision of health equipment and commodities, supervisory activities of CHNs with the goal of improving the quality of services, and facilitation of community participation in the community outreach programs in both the intervention and control areas. These measures might have been effective in reducing infectious diseases in children. In addition, intensified DHMT activities to prevent a cholera outbreak in the Ketu South District might have contributed to the significant reduction of diarrhea prevalence in both arms. There was a cholera outbreak in August 2014, and 595 cases were reported in the Ketu South District. The Volta Regional Health Directorate focused significant attention on the Ketu South District to prevent a new outbreak there and to avert the spread of cholera to neighboring districts. Accordingly, the Ketu South DHMT enhanced water, sanitation, and hygiene promotion. They started to distribute water purification tablets throughout the district after we launched the intervention, and also encouraged CHNs and community health officers (CHOs) to prioritize health education on water, sanitation, and hygiene during the community outreach program. Particularly, the DHMT emphasized the importance of boiling drinking water and food hygiene. In Togo, which borders Ghana along the Ketu South District, a cholera outbreak was reported in 2015 and 2016, which caused the Ketu South District to continue prioritizing water, sanitation, and hygiene promotion.

According to a recent systematic review, *Escherichia coli*, *Salmonella* spp., and *Streptococcus pneumonia* were the main pathogens of non-malaria febrile episodes among under-5 children in sub-Saharan Africa between 1990 and 2015, and some of those microbes are infectious agents of diarrhea [23]. We thus infer that the reduction in febrile episodes in both groups partly resulted from a reduction in infections with non-malarial agents, some of which are causative agents of both diarrhea and fever. We provided material incentives to all the respondents (e.g., soaps for the baseline survey and foodstuffs for the first and second follow-up surveys) to encourage them to actively participate in the survey, and we minimized the number of questions in our modified version of the Demographic and Health Surveys and Multiple Indicator Cluster Survey questionnaires to avoid respondent fatigue. Throughout the intervention period, households that reported diarrhea were given ORS and those that reported fever were tested for malaria, so we do not consider it likely that the respondents severely underreported diarrhea and fever in their children. However, we cannot rule out the possibility of a biased downward trend over time due to reporter fatigue over repeated measurements. During the course of follow-up, the registered children aged, and thus their diarrhea risk might have changed, which could be another reason for diarrheal reduction over time. We do not consider seasonal variation to have been a likely reason since the baseline and second follow-up surveys were both conducted during the same season (the rainy season). Likewise, we consider inter-observer bias between surveys to be a less likely explanation for our findings because the same group of data collectors was maintained throughout the survey. Our trial assumed a 25% prevalence of diarrhea and fever in the control group, and therefore it was likely underpowered to detect small to moderate effects.

Nevertheless, we found that the CHV intervention resulted in non-statistically significant reductions of 26% and 23% in diarrhea and fever prevalence at 12 months, which are in line with a recent meta-analysis of the effect of CHWs on child morbidity (RR 0.86, 95% CI 0.75–0.99) [6]. In addition, we found in an observational analysis that communities exposed to greater-intensity CHV intervention in terms of both duration and frequency of home visits at 6 months had significantly lower rates of diarrhea and fever than the control villages. These results suggest that the CHV intervention may reduce fever and diarrhea and that maintaining the fidelity of the intervention in terms of community coverage and duration of home visits is central to produce beneficial effects. Our findings that intervention fidelity and intensity modify the effect of the CHV intervention are similar to the results of a trial of CHWs and participatory women’s groups in India [9], where there was no overall effect of the intervention on child length-for-age *z*-scores or self-reported morbidity, but there was some indication of a greater effect among those exposed to higher-intensity interventions. As a result, it is essential to conduct research on how to best motivate and potentially incentivize unsalaried CHVs to reach and sustain high effective coverage of home visits.

The achieved coverage of CHV home visits at the community level was lower than we expected in the design stage, but it was similar to that of previous studies [10,24,25]. The proportion of the target population who had received a CHV home visit at least once in the previous 3 months was about 50%–60% throughout the study. The CHVs’ work burden in this trial design in terms of the minimum frequency and time of home visits was lower than in some other contexts [26,27] where a household typically received a 1-hour visit from a CHW every week or every other week. However, we had to reduce the work burden, taking into account the possibility of nationwide scaling up on a voluntary basis, since a high workload for CHWs is known to result in poor performance or lower motivation [28,29]. Still, the low coverage in this trial indicates that even this reduced home visit package was burdensome to CHVs, who likely had competing demands for income generation and family care. Similarly, the reduced duration of the home visits from 6 to 12 months also indicates that CHVs may have been losing their enthusiasm for the program over time, which could be explained by the lack of a monetary incentive system and their high work burden [3,4,18]. A previous study [24] stressed that if a CHV program is sustained, the loss of intensity will be compensated by repeated household visits with the passage of time. Stepwise capacity building of caregivers through multiple exposures to key messages by CHVs was the rationale for this argument [30]. However, we found that duration of the visit, and not the coverage, appeared to be important to produce benefit, and this raises the concern that merely exposing community members to more frequent visits might have no effect, even if the intervention continues. Reducing the CHV-to-population ratio [28,31] may allow CHVs to spend more time at each household.

This study also underscores the potential importance of optimizing the number of key messages to avoid message dilution. Indeed, a number of caregivers could not recall the full length of the messages they had received at both 6 and 12 months. Caregivers generally recalled 3 to 5 of the 10 key messages. Ghanaian policy states that CHWs must deliver comprehensive messages. Based on the findings of this study, 5 messages or fewer at each visit might be optimal for community members to recall; however, this issue warrants further study. It may not be effective for CHVs to deliver all the messages at every visit. Developing distinct sets of 5 or fewer key messages could be an alternative approach, enabling CHVs to deliver 1 set of messages during a certain time period, and when the awareness of people reaches a sufficient level communitywide, they could move on to the next set of messages. Having fewer messages may relieve the workload of CHVs and ultimately improve their performance. Gilmore and McAuliffe [11] argued that simple and targeted messages could improve the effectiveness of CHWs’ performance in health education. The importance of having CHVs equipped with communication and counseling skills cannot be overemphasized.

Our study has some limitations. As discussed previously, the trial may have been underpowered to detect small to moderate effects due to the large decline in diarrheal and fever prevalence in both the intervention and control group that was likely due to external factors. In addition, the possibility of information diffusion or spillover between the intervention and control groups cannot be ruled out. We documented that ~10% of households in the control communities reported receiving a CHV home visit every 3 months; however, caregivers may be misreporting CHN visits, which occurred in all study communities. In addition, this trial followed up participants for 1 year after the intervention, and therefore we were not able to investigate the longer-term effects of CHVs. In addition, caregivers’ practices were based on self-report, and as a result, the possibility of caregivers providing socially desirable responses or repeating key messages of the CHV program without implementing them cannot be excluded. Nevertheless, to minimize reporting bias, we also undertook direct observation of hygiene practices and mosquito net utilization.

Febrile episodes have been frequently used as an indicator of malaria, particularly in studies examining the prevalence of malaria in remote rural areas where diagnostic tests are hard to access [2,32]. We designed this study to use febrile illness as a proxy indicator of malaria prevalence instead of diagnostic test results. According to the *World Malaria Report 2018* by the World Health Organization, only a median of 52% of children with fever were reported to have been taken to a trained provider (i.e., to public sector health facilities, CHWs, or formal private health facilities), and a median of 40% of febrile children had not been taken to receive any form of care in 2015–2017 in 18 sub-Saharan Africa countries [2]. Among the children brought for care, 49% received a malaria diagnostic test (a finger or heel stick test) [2]. Our study also showed that only 21%–42% of children with febrile illness received an RDT for malaria, suggesting that diagnostic test results are not an adequate indicator of malaria prevalence.

However, febrile episodes also have limitations as an indicator of malaria prevalence. First, the symptoms of malarial fever are nonspecific [23]. The proportion of children with malaria among those with fever was reported to range from ≤10% to ≥70% in 2015–2017 in sub-Saharan Africa [33]. Similarly, Murphy and Breman estimated that only 30%–60% of children with fevers in Africa were actually infected with malaria [34]. Another limitation is recall bias. Fevers on the previous day at the time of the survey were more likely to be recalled than those that occurred a few days prior to the survey [35,36].

The first round of the survey to measure process indicators was conducted 3 months after the start of intervention implementation. Therefore, we suggest that the somewhat high percentage of healthy behaviors in handwashing, ITN use, and participation in the community outreach program at 3 months in part resulted from the program intervention. The baseline values of health behaviors were not assessed in the survey, and therefore were not adjusted for. Regardless of treatment allocation, CHNs, CHOs, and Community Health Management Committee (CHMC) members were trained in intervention delivery during the early period of the intervention. CHNs and CHOs are the frontline staff of Ghana’s health system based at health facilities and carrying out community outreach programs. Health education such as promoting handwashing and mosquito net utilization are the key components of community outreach programs. CHMC members are residents of their respective communities whose roles are to mobilize community members in community outreach programs and to advocate for health promotion, helping CHNs and CHOs. Not assessing process indicators at baseline is a limitation of this study because we cannot rule out the possibility of residual imbalance after randomization.

Unlike previous studies [37,38] conducted in Ghana, our findings suggest that CHVs may exert a beneficial effect on behaviors related to child health with high community coverage and regular household contacts of effective duration. Ghanaian health policy [39] articulates that it is mainly CHNs’ role to make home visits for counseling or health education. However, it may not be feasible for CHNs to conduct home visits on a regular basis, considering the large beneficiary population per CHN and logistical issues, such as the lack of transportation and the CHNs’ main role of caring for patients at CHPS health facilities. This study demonstrates the potential impact of CHVs in complementing CHNs’ critical tasks, to address the enormous shortage of the health workforce in Ghana and similar settings. Implementing CHV programs in a way that ensures frequent home visits of effective duration may prevent infectious diseases in children and contribute to achievement of global health and development goals.

## Supporting information

S1 Appendix Study protocol. (DOCX)

S2 Appendix Survey questionnaires. (DOCX)

S3 Appendix CONSORT checklist. (DOCX)

S1 Table Longitudinal GEE analysis of the effect of the CHV intervention on diarrhea, fever, malaria testing for fever, ORS for diarrhea, and family planning prevalence, at 6 and 12 months of follow-up. (DOCX)

S2 Table Recall of the 10 key messages of the CHV program at 6 and 12 months follow-up. (DOCX)

S3 Table Antenatal and prenatal care, and case management of malaria in pregnant women. (DOCX)

S1 Text CHV program outline. (DOCX)

## Abbreviations

CHMC: Community Health Management Committee
CHN: community health nurse
CHO: community health officer
CHPS: Community-based Health Planning and Services
CHV: community health volunteer
CHW: community health worker
DHMT: District Health Management Team
GEE: generalized estimating equation
ITN: insecticide-treated bed net
ORS: oral rehydration salts
RDT: rapid diagnostic test
RR: relative risk
