# Supplementary material for: Evaluating the impact of community health volunteer home visits on child diarrhea and fever in the Volta Region, Ghana: A cluster-randomized controlled trial
Source: PLoS Med. 2019 Jun 14;16(6):e1002830. doi: 10.1371/journal.pmed.1002830 (PMC6568387; doi:10.1371/journal.pmed.1002830)
Supplement: S1 Appendix — (DOCX) [file pmed.1002830.s001.docx]

**Effects of community health volunteers on infectious diseases of children under five in Volta Region, Ghana: study protocol for a cluster randomized controlled trial**

Yeonji Ma^1^, Heunghee Kim^2^, Yinseo Cho^1^, Jaeeun Lee^1^, Joseph Kwami Degley^3^, Abdul-Ghaffa Adam^4^, Gyuhong Lee^1^, Hoonsang Lee^1^, Seungman Cha^1,5*^

^1^ Korea International Cooperation Agency, 825 Daewangpangyo-ro, Sujeong-gu, Seongnam-si, Gyeongi-do, Republic of Korea; Emails: yeonjima@gmail.com (Ma Y); yinseocho@koica.go.kr (Cho Y); jelee21@koica.go.kr (Lee J); ghlee@koica.go.kr (Lee G); hoonsanglee.md@gmail.com (Lee H); jesusdongja@hanmail.net (Cha S)

^2^ Korea International Development Institute, 92 Saemunan-ro, Jongno-gu, Seoul, Republic of Korea; Emails: heungheek@gmail.com (Kim H)

^3^ Ketu South Municipal Health Directorate (Ghana Health Service), Aflao, Volta Region, P.O. Box 126, Republic of Ghana; Emails: joekwadeg@yahoo.com (Degley JK)

^4^ Devtplan Consult Limited., 212 Lame Dwaahe Street, Adenta-Accra, Republic of Ghana; Emails: abdulgafus@yahoo.com (Adam AG)

^5^ Department of Disease Control, Faculty of Infectious and Tropical Disease, London School of Hygiene & Tropical Medicine, Keppel Street, WC1E 7HT London, UK

Correspondence: [jesusdongja@hanmail.net](mailto:jesusdongja@hanmail.net), lsh127351@lshtm.ac.uk

**Abstract**

**Introduction:** In many low- and middle-income countries, community health volunteers (CHVs) are employed as a key element of the public health system in rural areas with poor accessibility. However, few studies have assessed the effectiveness of CHVs in improving child health in sub-Saharan Africa through randomized controlled trials. The present study aims to measure the impact of health promotion and case management implemented by CHVs on the health of under-5 children in Ghana.

**Methods and analysis:** This study presents the protocol of a cluster-randomized controlled trial assessing the impacts of CHVs, in which the community was used as the randomization unit. A phase-in design will be adopted, and the intervention arm will be implemented in the intervention arm during the first phase and in the control arm during the second phase. The key intervention is the deployment of CHVs, who provide health education, provide oral rehydration solutions and zinc tablets to children with diarrhea, and diagnose malaria using a thermometer and a rapid diagnostic test kit during home visits. The primary endpoints of the study are the prevalence of diarrhea and fever/malaria in children under 5 years of age, as well as the proportion of affected children receiving case management for diarrhea and malaria. The first and second rounds of household surveys to collect data will be conducted in the first phase, and the final round will be conducted during the second phase.

**Discussion:** With growing attention paid to the roles of CHVs as an essential part of the community health system in low-income countries, this study will contribute valuable information to the body of knowledge on the effects of CHVs.

**Ethics:** The study was approved by the Ghana Health Service Ethics Review Committee under the Ministry of Health, Republic of Ghana on July first, 2015.

**Trial registration number:** ISRCTN49236178. (June 16^th^, 2015)

**Keywords**

Community health workers, Community health systems, Diarrhea, Malaria, Child Health, Ghana

**Introduction**

Despite the substantial progress made between 1995 and 2015, over half of the deaths in children under 5 years of age in sub-Saharan Africa are still caused by malaria, diarrhea and pneumonia.^1^ Malaria alone caused 306,000 deaths among under-5 children in 2015 globally, including 292,000 deaths in sub-Saharan Africa.^2^ Diarrhea, largely due to the lack of safe water, sanitation and hygiene, killed 578,000 children in 2013, accounting for 9.2% of under-5 child deaths worldwide.^3^

Of the 62,000 deaths among Ghanaian children under 5 years of age in 2013, the majority were caused by malaria (20%), acute respiratory infections (13%), and diarrhea (8%).^4^ While other infectious diseases, such as HIV/AIDS and measles, have significantly decreased over the last decade in Ghana, diarrhea-specific child mortality has stagnated and malaria-specific deaths have decreased only by one percentage during the same period.^4^

Globally, given the limited human resources in the health sector, a community-based approach has been promoted as a cost-effective and pro-poor intervention to improve the accessibility of health care.^5, 6^ Community health workers (CHWs) are often employed as a key element of the community-based approach to the rural population of low- and middle-income countries.^7^ CHWs, in general, are defined as the non-professional lay health workers who are originally from the community and equipped with training and incentives to provide promotional, preventive, or curative health care services to the community members.^8-10^ They are known by various names in different settings, depending on the type of services offered, the compensation scheme, and the level of integration with the formal health system. For instance, Nigeria administers a village health worker program in which they are allowed to perform promotional work only, whereas health extension workers in Ethiopia are allowed to offer curative services for major illnesses. Lady health workers in Pakistan are paid as government employees, whereas a community health worker program in Tanzania operates based on volunteerism without regular payments.^11^

In Ghana, the Community-based Health Planning and Services (CHPS) initiative was adopted in 1999 as a national policy establishing a community health system to improve the accessibility and affordability of health care for residents in the remote areas.^6^ A distinctive feature of the CHPS system is the requirement of strong community engagement and participation. Community health nurses (CHNs) and community health volunteers (CHVs) are considered to be CHWs in the Ghanaian CHPS system. CHNs are formally trained for 2 years, bear the main responsibility for community health management within the formal governmental apparatus, and are on the government payroll. In contrast, CHVs are trained for a week and voluntarily support the outreach activities of CHNs and community participation, and are not paid by the government. Since community participation is a key aspect of the mechanism of the CHPS system, the role of CHV is as much critical as that of CHNs.^6, 12^

A number of studies have demonstrated that community-based health systems, particularly those involving CHWs, have led to improved access to maternal healthcare services^13-15^, modern contraception^16, 17^, and neonatal care among the poor.^18-22^ However, recently only few studies have investigated the impact of interventions made by CHWs on childhood illness in sub-Saharan Africa, using randomized controlled trials (RCTs).^23-26^ Moreover, many previous studies^27-31^ have evaluated the effect of CHWs only with the curative treatment while only one^26^ testing the effect with the preventive roles with RCT. A recent Cochrane systematic review evaluated 10 trials investigating the impact of CHWs with the expanded roles including prescription of anti-malarial drugs on the health of African children.^32^ Eight of them demonstrated the prescription of the drugs by CHWs without confirmation despite the World Health Organization’s recommendation to perform malaria diagnostic testing on all suspected malaria cases before administering treatment since 2010.^2^ To our knowledge, only one study published using RCT so far has tested the impact of CHWs in diarrhea management without provision of antibiotics while many others include the intervention with antibiotic prescription.^26^

Therefore, there is need for research testing the impact of CHWs, specifically regarding the promotive and preventive roles in major childhood illnesses in sub-Saharan Africa.

The study aims to measure the impact of CHVs on reducing childhood illnesses, especially malaria and diarrhea, in Ghana, which is one of the countries that has implemented a CHW program. We plan to explore the impact of CHVs, especially with regard to their role in the provision of health promotion, first-aid management for diarrhea oral rehydration solution (ORS) and zinc tablet, early diagnosis of malaria using rapid diagnostic tests (RDTs), and referrals for further treatment.

**Methods**

***Study setting***

The Ketu South district is one of the 25 administrative districts in the Volta Region, located in the southeastern corner of Ghana (See Figure 1). The Ketu South district is a relatively low land area, with altitudes ranging from less than 15 meters at the coast to 66 meters inland. The 2010 Population and Housing Census recorded a total population of 160,756, with 52.9% of the population consisting of females. The projected population for the district for 2015 based on an annual growth rate of 2.5% is 181,881, with 36,376 children under 5 years of age. The people of Ketu South are the Ewe tribe, which inhabits some parts of Togo, Benin, and the Volta Region of Ghana. They are a patrilineal society governed by a hierarchical, centralized authority. The district is culturally homogenous, with negligible variations. According to the 2010 Population and Housing Census, Christianity accounts for 59.0% of the total population, followed by traditional religion (27.9%) and Islam (3.5%). The people living in the district are predominantly fishermen, fish mongers, petty traders, and weavers of *kente* (a specific type of silk and cotton fabric that originated in the Ashanti Empire), with a few government workers interspersed among the population.

Figure 1. Study area.

| 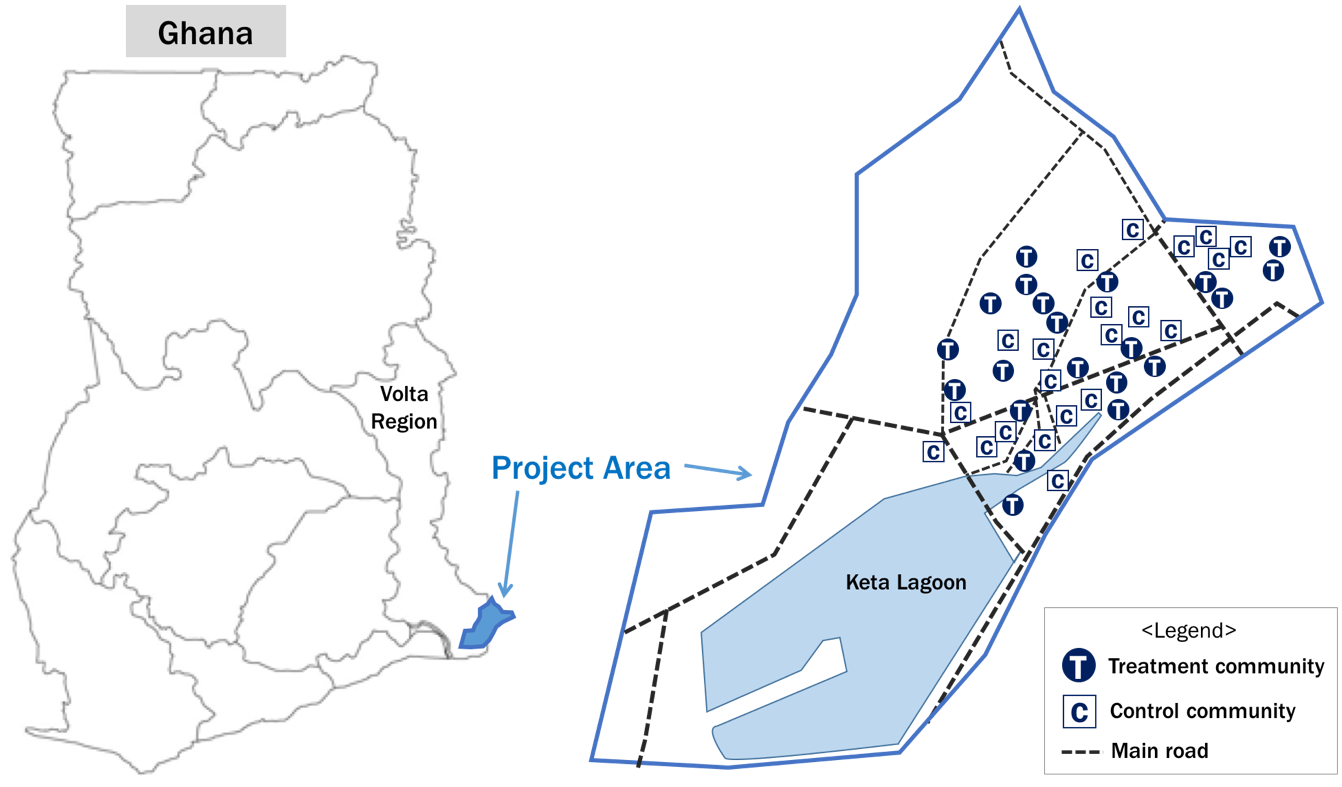 |
| --- |

***Study design***

In the study, the community was taken as the randomization unit, because we expected that CHV activation will impact disease prevention and treatment among households within a given community, which is where people interact with one another most closely. All interventions related to CHV activation will be performed at the level of each community. A phase-in design will be used for the study. CHVs will be activated in the intervention arm during the first phase, and a comparable intervention will be performed in the control arm during the second phase.

The study was approved by the Ghana Health Service Ethics Review Committee under the Ministry of Health, Republic of Ghana (Ethics approval ID: GHS-ERC:07/01/15), and registered as an international standardized RCT on June 16, 2015 (ISRCTN49236178/ http://www.isrctn.com/ISRCTN49236178).

***Primary endpoint***

This study aims to assess the effect of CHVs on multiple child health outcomes. The primary endpoints of the study are the prevalence of diarrhea and fever/malaria among children under 5 years of age, and the proportion of children receiving case management for diarrhea and malaria. We plan to use the 14-day prevalence of reported diarrhea and the 14-day prevalence of fever/malaria in the household as indicated in caregiver’s reports.

For the case management for diarrhea, we will investigate whether a child with diarrhea has been administered ORS. For malaria, we will measure whether a child with malaria symptoms was tested using an RDT kit and whether he or she was referred to a health facility if diagnosed with malaria.

The survey will be conducted three times at the household level: the first and second round will be carried out in the first phase, and the final round will be carried out in the second phase.

Diarrheal is defined as 3 or more instances of watery stools within 24 hours over the last 14 days. Febrile episodes will be assessed as a proxy indicator of malarial prevalence based on caregiver reports.

The main outcomes and indicators are presented in Table 1.

Table 1. Main outcomes and indicators.

| **Indicator (%)** (Denominators are within the respondents of the household survey) | | |
| --- | --- | --- |
| Use of ORS among under-5 children with diarrhea | Numerator | Number of U5C^1^ who took ORS^2^ when experiencing diarrhea over the past 14 days |
|  | Denominator | Number of U5C who experienced diarrhea in the past 14 days |
| Prevalence of diarrhea in under-5 children | Numerator | Number of U5C who experienced diarrhea in the past 14 days |
|  | Denominator | Number of U5C |
| Diagnosis rate of malaria in under-5 children using an RDT | Numerator | U5C diagnosed with RDT for malaria in the last two weeks |
|  | Denominator | U5C who have complained of fever in the last two weeks |
| Malaria incidence in under-5 children | Numerator | U5C who have complained of fever in the last two weeks |
|  | Denominator | Number of U5C |

^1^ U5C, under-5 children; ^2^ ORS, oral rehydration solution

***Intermediate indicators***

Intermediate indicators for the prevalence of diarrhea in children include the hand-washing behaviors of caregivers at 4 critical times (before cooking, after defecating, before feeding the child, before eating). An intermediate indicator for child malarial prevalence is the utilization of insecticide bed nets the previous night while sleeping.

***Process indicators***

We are going to carry out a process evaluation along with the impact evaluation to investigate dose delivered and dose received. First, logbooks will be recorded by recorded by CHVs themselves and signed by caregivers, where we can assess how many key messages have been delivered at each household level. Second, four rounds of household survey will be conducted, with which we will measure how many and what key messages caregivers could recall.

***Sample size calculation***

Based on a preliminary survey in 2013, we estimated that the malaria prevalence was 25% and assumed that the prevalence would be reduced by our intervention by 25%, based on systematic reviews.^33^ Assuming a coefficient of variation of 0.16, a 10% loss to follow-up, a study power of 80%, a 25% prevalence of malaria in the absence of an intervention, and a 25% relative decrease after the intervention resulted in the need for 20 clusters and 950 household per arm, using a formula published elsewhere.^34^

***Household sampling methods***

The demographic information of each community in the Ketu South district was compiled by the health directorate through reports by CHNs in each CHPS zone prior to the baseline survey. The total number of communities in the Ketu South district was 57, and we applied the probability-proportionate-to-size method to select 40 communities (See Figure 2). We excluded urban area for the trial since CHV may operate differently between urban and rural areas.

***Eligibility criteria and randomization***

Forty communities were assigned into 8 strata on the basis of the prevalence of diarrhea in under-five children, economic status of the community, and the proportion of mothers who delivered their youngest child by a skilled birth attendant based on the results of the first-round survey. To evaluate the socioeconomic status of a community, we used the proportion of households with wattle and daub houses as representatives of the lowest economic level. We ensured that an equal number of communities within all 8 strata were randomly allocated into the intervention and control arms, with each arm including 20 communities. Microsoft Excel version 15.22 was used for randomization. We ensured comparability in the main outcomes between the intervention and control arms by strictly applying randomization. After conducting the first-round survey, we classified the clusters into strata by primary endpoint and socioeconomic status. In doing so, we were able to ensure a high comparability and therefore were able to avoid selection bias. Control communities are allocated by randomization among the target communities. We found control and intervention communities well balanced in terms of caregivers’ demographic and socioeconomic status, and child demographic status and several key variables related to primary outcomes of the intervention (Table 2).

Figure 2. Flow diagram of the trial.

| 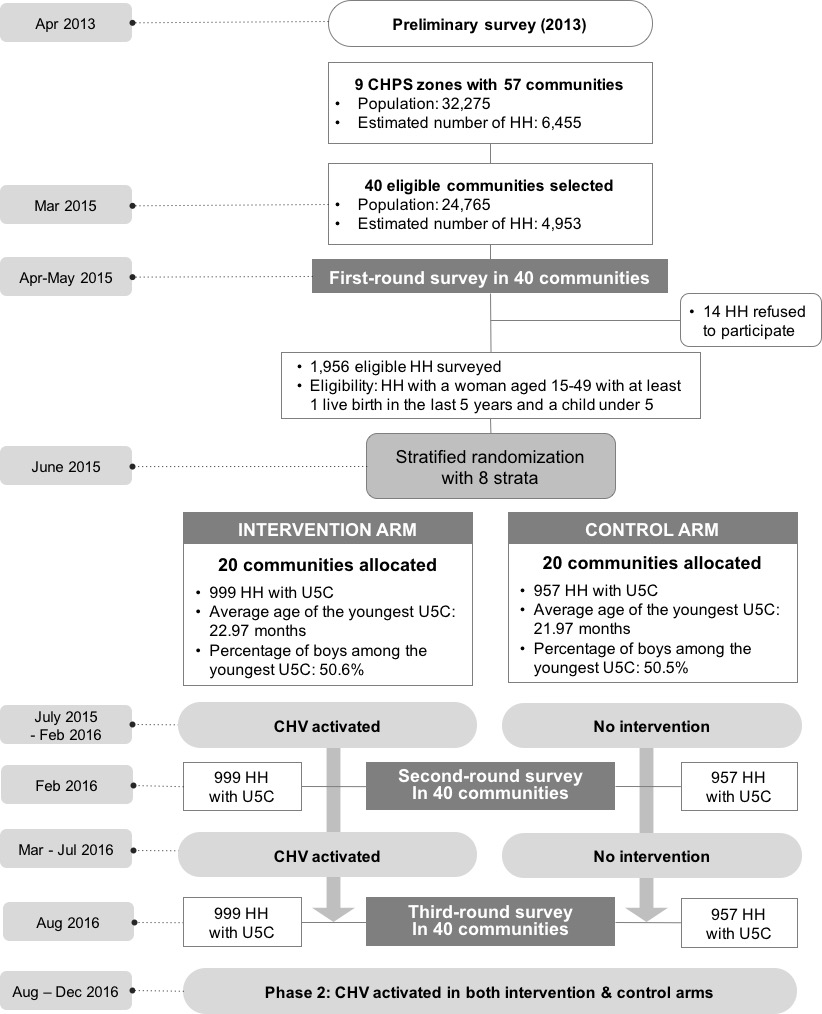 |
| --- |

Table 2. Results of the first-round survey in the intervention and control arms.

| **Variables** | | **Intervention** | | **Control** | |
| --- | --- | --- | --- | --- | --- |
|  |  | N | % or  mean (SD^2^) | N | % or  mean (SD) |
| Caregiver's age (years) | | 999 | 29.00(7.16) | 957 | 28.86(6.78) |
| Religion of respondent | Christian | 614 | 61.50% | 599 | 62.60% |
|  | Traditionalist | 280 | 28.00% | 256 | 26.80% |
| Education level of respondent | Graduated basic school | 188 | 23.6% | 165 | 22.5% |
|  | Dropped out of basic school | 455 | 57.2% | 406 | 55.3% |
|  | Graduated secondary school | 19 | 2.4% | 24 | 3.3% |
| Possession of NHIS^1^ Card | Have a valid card | 332 | 33.2% | 328 | 34.3% |
| Age of the youngest child under-5 child (In months) | | 989 | 22.97 (15.95) | 946 | 21.97 (15.94) |
| Youngest child’s sex | Male | 505 | 50.6% | 483 | 50.5% |
| Main source of drinking water for the last-born child | Hand-dug well | 268 | 26.8% | 248 | 25.9% |
|  | Sachet water | 202 | 20.2% | 223 | 23.3% |
|  | Borehole | 190 | 19.0% | 141 | 14.7% |
|  | Bottled water | 163 | 16.3% | 150 | 15.7% |
|  | Public standpipe | 70 | 7.0% | 96 | 10.0% |
| Possession of Child Health Record Book | | 689 | 69.0% | 671 | 70.1% |
| Most recently measured weight of the last-born child | Average weight (kg) | 689 | 9.14(2.60) | 671 | 8.92(2.66) |
| Diarrhea incidence in the last-born child | | 175 | 17.5% | 192 | 20.1% |
| Provision of ORS to the last-born child having diarrhea | | 94 | 53.7% | 97 | 50.5% |
| Febrile episode in the last-born child | | 294 | 29.4% | 337 | 35.2% |
| Child had malaria test when febrile | | 55 | 17.7% | 57 | 16.9% |

^1^ NHIS: National Health Insurance Scheme; ^2^ SD: standard deviation.

**Eligibility**

Households with one or more under-5 children were eligible for the trial. Households were excluded from the first-round survey if a caregiver declined to participate in the survey. Enrolled households were registered with a distinctive identification code at the time of the first-round survey and will be followed for the next 12 months of the trial period.

***Key intervention***

Our key intervention is to activate CHV activities, and the intervention period is 1 year. The main duty of CHVs is to pay regular visits to 40 assigned households in their community and to carry out key activities mainly targeting children under five and caregivers. CHVs are required to visit 20 households every month so each participating family can receive a visit every other month. During home visits, CHVs conduct health education with key messages on sanitation and hygiene to prevent diarrhea and other infectious diseases such as hand-washing, management of diarrhea using ORS or recommended home-maid fluid, prevention of malaria including utilization of insecticide-treated nets, nutrition, breastfeeding and so on. CHVs also provide services for under-5 children to manage diarrhea with ORS and zinc and to diagnosis malaria using a thermometer and an RDT kit. CHVs will be requested to deliver all key messages applicable to the household on every home visit.

CHVs will be nominated by community committees on the basis of their literacy, volunteerism, and working experience as a volunteer in health-related fields with government, donor agencies, or non-governmental organizations. The selection criteria are based on the national CHPS guidelines, and the District Health Management Team coordinated the selection process.

The training curriculum for CHVs will be developed in reference to CHV’s training manual^35^, as recommended in the CHPS implementation guidelines. District and regional health staff will provide 5 days of training. During the 5-day training program, CHVs will learn about the concept of CHPS, their roles and responsibilities, communication skills with community members, and will gain a basic knowledge of essential maternal and child health care services as well as reporting and recording skills. After the initial training, two types of refresher training will be given. One will involve monthly meetings at the CHPS compounds with flexible topics based on the CHV’s request, and the other is a workshop to be conducted every 6 months.

A backpack, t-shirts, an identification card, a CHV certification, a digital thermometer, and stationery will be provided to CHVs. Health equipment such as ORS packets, zinc tablets, and RDT kits will be dispensed in a timely manner to assist their activities. Limited material incentives of 8.7 USD (30 CHC) worth of food items and 1.5 USD (5 GHC) worth of airtime will be also provided every month. *(The exchange rate on August 1, 2015 was applied: 1USD = 3.46 GHC.)*

The first phase, in which CHVs will be deployed in the 20 treatment communities, will last for one year.

We referred to the suggested roles in the latest CHPS implementation guidelines (draft version) and the recommended training manual for CHV training^35^ in order to specify the roles of CHVs. Table 3 summarizes the responsibilities of CHVs in the trial. The key role of CHVs is to perform home visits to assigned households in their village, and they are also required to support the community health officers (CHOs) and CHNs, to surveil major health events, to mobilize community members, and to comply with regular supervision and training.

It is critical for this trial to ensure that CHVs are properly activated in the intervention communities. The key indicators for measuring the activation level of CHVs are the frequency of their home visits, their participation in the monthly meetings, and their retention rate (Table 4).

Table 3. Job description for community health volunteers.

| **Category** | **Descriptions** |
| --- | --- |
| 1. Home visit | - Pay a home visit (minimum) once per every two months to 35 assigned households - Provide health education to the mothers and family members through constant delivery of key messages mainly regarding the prevention and management of child illnesses, family planning, hygiene and sanitation, antenatal care, postnatal care, and skilled delivery - Provide diagnostic services in malaria to under-5 children and pregnant women using a thermometer and RDT^1^ kit - Provide ORS and zinc tablets for the primary management of diarrhea in under-5 children - Provide Aquatabs to households without an appropriate source of drinking water - Provide family planning counseling to key decision-makers in the family - Support healthy behaviors of the community members (i.e. help setting ITNs^2^, referring clients to a corresponding health facility) |
| 2. Support for CHOs/CHNs | - Support child welfare clinic (CWC^3^) of CHOs^4^/CHNs^5^ in outreach and mobilize the community - Support CHOs/CHNs in referral and management of clients |
| 3. Surveillance | - Record logbook - Record Community Register for Vital Health Events - Find and report pregnancies and newborn cases |
| 4. Community mobilization and sensitization | - Participate in the community meetings and committees and raise health issues - Organize health talks at community gatherings |
| 5. Reporting and training | - Attend an initial training session for 5 days - Attend 6-month workshop sessions - Participate in monthly meeting at CHPS^6^ and report on monthly activities (a refresher session is planned as a part of the monthly meeting) - Manage the provided supplies and submit a report regarding their use |

^1^ RDT: rapid diagnostic test, ^2^ ITN: insecticide-treated net, ^3^ CWC: child welfare clinic, ^4^ CHO: community health officer, ^5^ CHN: community health nurse, ^6^ CHPS: Community-based Health Planning and Services

Table 4. Indicators for measuring the activation of community health volunteers.

| **Component** | **Indicator** | **Measurement tool** |
| --- | --- | --- |
| Service delivery | The percentage of CHVs who have visited all the assigned households (20) in the last month (among those retained) | CHV logbook at monthly meeting |
| Participation in monitoring | The percentage of CHVs who participated in the monthly meeting at CHPS more than two-thirds of the whole year | Monthly meeting minutes |
| Retention | The percentage of CHVs retained | CHO records on CHV supervision |

***Project implementation***

The Improving Maternal and Child Healthcare project in Volta Region of Ghana is being conducted in the Keta, Ketu South and Ketu North districts of from January 2014 to December 2016. The 3 project districts have a total of 24 CHPS facilities within 132 CHPS zones, with a population of 461,939, including 92,387 children under the age of 5 years. This project aims to enhance the accessibility, availability, and quality of the health services for mothers and under-5 children. In order to accomplish this goal, it provides equipment to health facilities, deploy and activate CHVs, and promotes public awareness.

The project is funded by the Korea International Cooperation Agency and implemented by a consortium of the Graduate School of Public Health, Yonsei University and the Korea International Development Institute.

***Data analysis***

Intention-to-treat analysis will be conducted to explore the effect of CHV activation on childhood infectious disease of under-five children. For diarrheal prevalence as a dependent variable, water quantity and quality, hygiene behavior of caregivers, presence of improved latrines, characteristics of the enrolled child such as age and sex, household income, and caregiver’s education level will be controlled for in quantifying the net effect of CHVs. For malaria prevalence as a dependent variable, the enrolled child’s characteristics, household income, and caregivers’ education level will be controlled. Environmental risk factors such as seasonality and altitude are also considered to be independent variables for malaria prevalence. However, the intervention and control arms in our study site present no difference in those factors so there will be no control for environmental factors. When quantifying the net effect of CHVs on child malaria prevalence, insecticide-treated bed net utilization will not be controlled since it is considered an intermediate factor between intervention and disease outcome. Generalized estimating equations will be used for investigation at the cluster level. The random effects model will be used, taking account of between-cluster variation by assuming that there are cluster-level effects. Per-protocol analysis will be performed to obtain additional background information if the overall percentage of CHVs activated does not reach 80%. Multi-level analysis will also be conducted to explore whether the effects of CHVs on child diarrheal and malaria prevalence vary by community depending on a community’s overall coverage of activated CHVs.

**DISCUSSION**

This study seeks to assess the effects of CHVs on the health gains of children under 5 years of age. We thus rigorously applied a randomization process after calculating adequate cluster and household sample sizes.

In this study, we place an emphasis on the preventive role of CHVs in child health. The role of CHVs is to provide education about hand-washing, breastfeeding, the utilization of insecticide-treated nets, ORS treatment and rapid malaria tests, as well as making referrals for sick children to health facilities. We expect the health education using key messages during home visit will facilitate their function for disease prevention. Considering the context of resource-poor settings, the public health implications of the preventive role of CHVs is not trivial.

Previous studies did not describe the nature of CHV training or the availability of further education after the initial course. In this trial, CHVs are trained one week at the start of the project and regular training will be provided on a monthly basis by CHOs and CHNs.

We performed a pre- and post-test right before and after the CHV training. If we found a CHV not achieving a score of 80 out of 100, we retrained him or her. To standardize the regular training, standard operating procedure on the implementation of monthly meetings will be provided to the supervisors of the CHVs and it includes the guidance on the training. The regular training will be conducted based on the topics and messages from the home visit booklet that CHVs were provided with to facilitate their home visit education. Also, the monitoring team, composed of the district health directorate members and the project manager and staff, who oversee the monthly meeting and training activities of all CHVs, will coordinate the content and quality of regular training through active review and discussion.

The intervention is not restricted to health education. According to the operational definition of this trial, the key intervention is to help CHVs perform their duties, which mainly involve visiting at least 20 households each month to deliver key messages and provide basic health services established by the policy such as malaria diagnosis and ORS provision.

Through in-depth discussion with government officials and community leaders during project design, we realized that merely educating CHVs was not sufficient to help them perform their tasks. We thus developed active supervision and support from government officials such as CHPS in close collaboration with DHMT. By employing a phase-in design for the trial, the government made an agreement that CHV activation would be delayed in the control arm during the first phase since it could be carried out during the second phase. In this regard, we believe that contamination will not take place severely.

No monetary subsidies will be provided to CHVs during the trial to strictly comply with the policy of the Ghanaian government, according to which CHVs function as a bridge between community members and CHNs “without affecting the national wage bill,” and rely on the discretion of community members.

One of the strengths of this trial is that a process evaluation will be conducted in parallel with the impact evaluation. Few studies have investigated the impact of CHWs using process indicators. In this study, we will use process indicators at each stage to assess reliability, services delivered, services received, participation, recruitment, retention and quality. A range of data will be collected and analyzed to evaluate each process: the appropriateness of selection of CHVs, the quality of their training, the frequency of their visits to each household every two months, the number of key messages delivered to each household, the number of key messages caregivers can recall, community participation in relation to CHV activities, their retention rate, and changes in preventive and health-seeking behaviors. Implementation of process evaluation will ensure that we explore how the entire health system affects the activation of CHVs in terms of supervision, logistics, incentives, and by what mechanism this occurs. Four rounds of a household survey will be administered to collect data regarding the project process, where we will assess the extent to which CHVs will be supported by the community nurses and the entire health system as a whole.

One of the limitations of this trial is that malaria cases are not being confirmed using laboratory tests. We will measure fever reported by a caregiver as a proxy indicator of malaria prevalence in both arms of the trial. Although RDTs will be used to test for malaria in children with a fever, this will only be the case for households within the intervention arm since the testing will be conducted by CHVs. Despite its seasonal variance, fever, as the primary manifestation of malaria in young children, can be utilized as an indicator of malaria prevalence, although some source has suggested that it must be interpreted with caution [36].

The project team must start recruiting CHVs in control arm during the second phase and also reached an agreement with the local governments to commence CHV activation without any further delay right after the first phase regardless of any pronounced effects on children. If we have to implement CHV activation in the control arm, we cannot maintain two different arms with and without intervention, which means we cannot compare the outcomes of the intervention during this phase. Hence, we ruled out an optimization phase. We thus cannot consider an optimization phase for this trial although we think optimization is important.

Due to the absence of demographic data for the study district, we used estimated population figures for each community derived by the officials of the Ghana Health Service. However, during the baseline survey we found discrepancies between the estimated and actual number of households in communities, and these figures were adjusted during the sampling process in some communities. Although a pre-survey listing the households in each community is recommended to enhance the accuracy of household sampling, budgetary constraints prevented us from carrying out such a survey.

CHWs are expected to offer quality health services to the population at a reduced cost. To that end, CHWs have been often required to perform extensive duties, such as health extension workers in Ethiopia or CHWs in Kenya.^11, 37^ However, the quality of care may be harmed when CHWs are overloaded or given complicated tasks with only limited training.^37^ CHVs in Ghana are voluntary CHWs who are given a short period of training and a limited range of responsibilities, focusing on promotional and preventive health services. Therefore, this study exploring the effects of CHVs on child health will provide useful information regarding the adoption of voluntary CHW programs in sub-Saharan Africa.

As incased attention has been paid to the role of CHWs in community health systems in low-income settings, the roles, incentives, and training program involved have become increasingly diversified, which hinders a clear assessment of the effects of different types of CHW programs.^10^ This study will be a step forward in specifically testing the effectiveness of voluntary CHW cadres.

**Declarations**

**Acknowledgements** The authors thank participating community members, and Ghana Health Service.

**Funding** This project is funded by the Korea International Cooperation Agency (KOICA). The content of this publication is the responsibility of the authors and do not reflect the views of KOICA.

**Availability of data and materials** Study materials and data will be held by the KOICA. All study materials and data-sets are available on request from the corresponding author.

**Consent for publication** Not applicable.

**Ethics approval and consent to participate** Ethical approval to conduct the study has been obtained from the Ghana Health Service Ethics Review Committee (reference number GHS-ERC:07/01/15). The trial is currently being conducted according to good research practice and we obtained written informed consent from the participants by informing participants about the study and by asking for their informed consent to participate.

**Competing interests:** The authors declare that they have no competing interests. Adam AG declared that he had no competing interests in a written form.

**Data sharing statement:** In addition to the paper and the protocol, the informed consent documents and all the report forms are available on request.

**Contributors:** Ma Y, Cho Y, Lee J and Cha S conceived the study, and Ma Y and Cha S led the study design. Kim H and Degley JK provided expertise in the trial of the implementation of community health volunteers. Cho Y, Lee G, Lee J, Adam AG and Lee H participated in the design of the study and the implementation of the trial, and edited the manuscript. Ma Y, Lee J, Cho Y and Cha S drafted the manuscript. Cha S provided expertise on cluster-randomized trials. All authors read and approved the final manuscript.

**References**

1. UN: The Millennium Development Goals Report 2015. New York: United Nations; 2015. P.32-33.
2. WHO: World Malaria Report 2015. Geneva: World Health Organization; 2015.
3. Liu L, Oza S, Hogan D, Perin J, Rudan I, Lawn JE, et al. Global, regional, and national causes of child mortality in 2000-13, with projections to inform post-2015 priorities; an updated systematic analysis. Lancet. 2015;385: 430-40.
4. WHO. World Health Statistics 2015. Geneva: World Health Organization; 2015.
5. Adam MB, Dillmann M, Chen M-K, Mbugua S, Ndung’u J, Mumbi P, Waweru E, Meissner P: Improving Maternal and Newborn Health: Effectiveness of a Community Health Worker Program in Rural Kenya. PLoS ONE. 2014;9:e104027–8.
6. Nyonator FK, Awoonor-Williams JK, Phillips JF, Jones TC, Miller RA: The Ghana Community-based Health Planning and Services Initiative for scaling up service delivery innovation. Health Policy and Planning. 2005;20:25–34.
7. Lehmann U, Sanders D: Community health workers: What do we know about them? The state of evidence on programs, activities, costs and impact on health outcomes of using community health workers. Geneva: World Health Organization; 2007.
8. Pallas SW, Minhas D, Pérez-Escamilla R, Taylor L, Curry L, Bradley EH. Community health workers in low-and middle-income countries: what do we know about scaling up and sustainability?. American journal of public health. 2013;103(7):e74-82.
9. Bhattacharyya K, Winch P, LeBan K, Tien M. Community Health Worker Incentives and Disincentives: How They Affect Motivation, Retention, and Sustainability. Arlington, VA: Basic Support for Institutionalizing Child Survival Project (BASICS II) for the United States Agency for International Development; 2001.
10. Bhutta ZA, Lassi ZS, Pariyo G, Huicho L. Global Experience of Community Health Workers for Delivery of Health Related Millennium Development Goals: A Systematic Review, Country Case Studies, and Recommendations for Integration into National Health Systems. Geneva, Switzerland: Global Health Workforce Alliance and World Health Organization; 2010.
11. Community Health Framework: Distilling decades of Agency experience to drive 2030 Global Goals (Version 1.0). Washington: USAID; 2015.
12. National CHPS Policy: Accelerating attainment of Universal Health Coverage and bridging the access inequity gap. Accra: Ministry of Health Ghana; 2014. https://s3.amazonaws.com/ndpc-tatic/CACHES/PUBLICATIONS/2016/04/16/National+Community+Health+Planning+and+Services+Policy.pdf. Accessed 10 Aug 2016.

Sakeah E, Doctor HV, McCloskey L, Bernstein J, Yeboah-Antwi K, Mills S. Using the community-based health planning and services program to promote skilled delivery in rural Ghana: socio-demographic factors that influence women utilization of skilled attendants at birth in Northern Ghana. BMC Public Health. 2014;14:1.

1. Viswanathan K, Hansen PM, Rahman MH, Steinhardt L, Edward A, Arwal SH, Peters DH, Burnham G: Can community health workers increase coverage of reproductive health services? Journal of Epidemiology & Community Health. 2012;66:894–900.
2. Mullany LC, Lee TJ, Yone L, Lee CI, Teela KC, Paw P, Shwe Oo EK, Maung C, Kuiper H, Masenior NF, Beyrer C. Impact of Community-Based Maternal Health Workers on Coverage of Essential Maternal Health Interventions among Internally Displaced Communities in Eastern Burma: The MOM Project. PLoS Med. 2010;7:e1000317–11.
3. Douthwaite M, Ward P. Increasing contraceptive use in rural Pakistan: an evaluation of the Lady Health Worker Programme. Health Policy and Planning. 2005;20:117–123.

Debpuur C, Phillips JF, Jackson EF, Nazzar A, Ngom P, Binka FN: The Impact of the Navrongo Project on Contraceptive Knowledge and Use, Reproductive Preferences, and Fertility. Studies in Family Planning. 2002;33:141-164.

1. Penfold S, Manzi F, Mkumbo E, Temu S, Jaribu J, Shamba DD, Mshinda H, Cousens S, Marchant T, Tanner M, Schellenberg D, Schellenberg JA. Effect of home-based counselling on newborn care practices in southern Tanzania one year after implementation: a cluster-randomised controlled trial. BMC Pediatrics. 2014;14:187.
2. Quayyum Z, Khan MNU, Quayyum T, Nasreen HE, Chowdhury M, Ensor T. “Can community level interventions have an impact on equity and utilization of maternal health care” – Evidence from rural Bangladesh. International Journal for Equity in Health. 2013;12:22.
3. Lewycka S, Mwansambo C, Rosato M, Kazembe P, Phiri T, Mganga A, Chapota H, Malamba F, Kainja E, Newell M-L, Greco G, Pulkki-Brännström A-M, Skordis-Worrall J, Vergnano S, Osrin D, Costello A. Articles Effect of women’s groups and volunteer peer counselling on rates of mortality, morbidity, and health behaviours in mothers and children in rural Malawi (MaiMwana): a factorial, cluster-randomised controlled trial. The Lancet. 2013;381:1721–1735.
4. Tomlinson M, Doherty T, Ijumba P, Jackson D, Lawn J, Persson LÅ, Lombard C, Sanders D, Daviaud E, Nkonki L, Goga A, Rohde S, Sitrin D, Colvin M, Chopra M. Goodstart: a cluster randomised effectiveness trial of an integrated, community-based package for maternal and newborn care, with prevention of mother-to-child transmission of HIV in a South African township. Tropical Medicine & International Health. 2014;19:256–266.
5. Kumar V, Mohanty S, Kumar A, Misra RP, Santosham M, Awasthi S, Baqui AH, Singh P, Singh V, Ahuja RC, Singh JV, Malik GK, Ahmed S, Black RE, Bhandari M, Darmstadt GL. Effect of community-based behaviour change management on neonatal mortality in Shivgarh, Uttar Pradesh, India: a cluster-randomised controlled trial. The Lancet. 2008;372:1151–1162.
6. Pence B, Phillips J, Debpuur C: The Effect of Community Nurses and Health Volunteers on Child Mortality: The Navrongo Community & Family Planning Project. Population Council Working Paper 2005; 200:1-20.
7. Kidane G, Morrow RH. Teaching mothers to provide home treatment of malaria in Tigray, Ethiopia: a randomised trial. Lancet. 2000;356(9229):550–5.
8. Kouyate B, Some F, Jahn A, Coulibaly B, Eriksen J, Sauerborn R, et al. Process and effects of a community intervention on malaria in rural Burkina Faso: randomized controlled trial. Malaria Journal. 2008;7:50.
9. Brenner JL, Kabakyenga J, Kyomuhangi T, Wotton KA, Pim C, et al. Can Volunteer Community Health Workers Decrease Child Morbidity and Mortality in Southwestern Uganda? An Impact Evaluation. PLoS ONE. 2011;6(12):e27997. doi:10.1371/journal.pone.0027997.
10. Christopher JB, Le May A, Lewin S, Ross DA. Thirty years after Alma-Ata: a systematic review of the impact of community health workers delivering curative interventions against malaria, pneumonia and diarrhoea on child mortality and morbidity in sub-Saharan Africa. Hum Resour Health. 2011;9:27. doi: 10.1186/1478-4491-9-27.
11. Menon A, Snow RW, Byass P, Greenwood BM, Hayes RJ, N’jie AB. Sustained protection against mortality and morbidity from malaria in rural Gambian children by chemoprophylaxis given by village health workers. Transactions of the Royal Society of Tropical Medicine and Hygiene. 1990;84:768-772.
12. Hill AG, Macleod WB, Joof D, Gomez P, Walraven G: Decline of mortality in children in rural Gambia: the influence of village-level primary health care. Trop Med Int Health. 2000;5:107-18.
13. Greenwood BM, Greenwood AM, Bradley AK, Snow RW, Byass P, Hayes RJ, N’jre AB. Comparison of two strategies for control of malaria within a primary health care programme in The Gambia. Lancet. 1988;1:1121-1127.
14. Alonso PL, Lindsay SW, Armstrong JR, Conteh M, Hill AG, David PH, Fegan G, De francisco A, Hall AJ, Shenton FC. The effect of insecticide-treated bed nets on mortality of Gambian children. Lancet. 1991;337:1499-502.
15. Okwundu CI, Nagpal S, Musekiwa A, Sinclair D. Home‐or community‐based programmes for treating malaria. The Cochrane Library. 2013;5.
16. Lewin SA, Babigumira SM, Bosch-Capblanch X et al. Lay health workers in primary and community health care for maternal and child health and the management of infectious disease. Cochrane Database of Systematic Reviews 2010, Issue 3. Art. No.:CD004015.
17. Hayes RJ, Bennett S. Simple sample size calculation for cluster-randomized trials. Int J Epidemiol. 1999;28:319–26.
18. The Population Council, ICN., Ministry of Health/Ghana Health Service. Community-based Health Planning and Services (CHPS): Community Health Volunteer's Training Manual. 2009.
19. Ghana Statistical Service(GSS), Ghana Health Service(GHS), and ICF International. Ghana. Demographic and Health Survey 2014. Rockville, Maryland, USA: GSS, GHS, and ICF International; 2015. p.135-136.
20. Fulton BD, Scheffler RM, Sparkes SP, Auh EY, Vujicic M, Soucat A. Health workforce skill mix and task shifting in low income countries: a review of recent evidence. Hum Resour Health. 2011;11:9:1. doi: 10.1186/1478-4491-9-1.
