# Supplementary material for: Evaluating the impact of community health volunteer home visits on child diarrhea and fever in the Volta Region, Ghana: A cluster-randomized controlled trial
Source: PLoS Med. 2019 Jun 14;16(6):e1002830. doi: 10.1371/journal.pmed.1002830 (PMC6568387; doi:10.1371/journal.pmed.1002830)
Supplement: S2 Appendix — (DOCX) [file pmed.1002830.s002.docx]

**MATERNAL AND CHILD HEALTH SURVEY- VOLTA REGION**

questionnaire for individual womAn

| **general Information/ introduction GI**  **THIS QUESTIONNAIRE IS TO BE ADMINISTERED TO WOMEN AGE 15-49 YEARS WITH AT LEAST ONE BIRTH IN THE PAST FIVE YEARS**  if both answers are yes, then start survey.   - are you a woman age 15-49? - have you given birth to at least one child in the past five years? | |
| --- | --- |
| good morning/afternoon/evening. my name is ……….I am a part of team undertaking a data collection exercise in this community on behalf of korea international cooperation agency (koica). koica is implementing a project to improve maternal and child health status in volta region. we are conducting a survey about the health situation of mothers, children and families. i will be grateful if you will permit me to have a discussion on these issues with you now and follow up interviews on same issues in the near future. you are under NO obligation to take part in the interview. However if you choose to participate all the information we obtain will remain strictly confidential and anonymous under any circumstances. I assure that the information you give will be solely used for the purpose of the project. the interview will take about one hour. | |
| Do you agree to participate?   - *Yes, permission is given* ⇨*Go to GI1 to begin the interview.* - *No, permission is not given* ⇨*Thank her and End interview.* | |
| SIGNATURE OF INTERVIEWER: | Date: |

| **GI1.** Sub-district/Area Council: | |
| --- | --- |
| **GI2.**: Community Name: | **GI3**. Cluster number: |
| **GI4.** House Number: | **GI5.** House Address: |
| **GI6.** Household head’s Name: | **GI7.** Woman’s Name: |
| **GI8**. Woman’s Survey Number**:** | **GI9.** Woman’s Telephone Number: |
| **G20.** Housing Type  Sancrete………………...1  Land crete ……………...2  Bricks…………………...3  Wattle and daub………...4 |  |

| **G110.** Interviewer’s name: | **GI11**. Interviewer ID |
| --- | --- |
| **GI12**. Interviewer Tel No | **GI13.** Day / Month / Year of Interview |

| Household BACKGROUND HB | | |
| --- | --- | --- |
| **HB1**. How old are you?  *Probe: Which year were you born?*  If she cannot answer properly, move to WB2*.* | Age (in completed years) __ __  DK ……………………………………….99 |  |
| **HB2**. Your age range? | 15-19 01  20-24 02  25-29 03  30-34 04  35-39 05  40-44 06  45-49 07 |  |
| **HB3.**What is your current marital status | Single 01  Married 02  Cohabited 03  Divorced 04  Separated 05  Widowed………………………………………06 | If 2,4,5,6 Skip HB9 and HB10 |
| **HB4.** What is your religious affiliation | Christian 01  Moslem 02  Traditionalist 03  No religion 04  Others, specify ………………………………… |  |
| **HB5**. Have you ever attended school? | Yes 1  No 2 | 2⇨HB7 |
| **HB6**. What is your highest level of education? | None 01  Preschool drop-out 02  Preschool graduated 04  Basic School drop-out 05  Basic School graduated 06  Secondary drop-out 07  Secondary graduated 08  Tertiary drop-out 09  Tertiary graduated 010  Others, specify …………………………………. |  |
| **HB7.** Are you employed (ie engaged in any economic activity for pay or profit) | Yes 1  No 2 | 2⇨HB9 |
| **HB8.** If employed, kindly give details | Employee (Government)………………………01  Employee (Private sector)……………………..02  Self-employed (Farming/fishing)…….………..03  Self-employed (commerce)……………………04  Others, specify………………………………… |  |
| **HB9.** Is your husband/partner employed (ie engaged in any economic activity for pay or profit) | Yes 1  No 2 | 2⇨HB11 |
| **HB10.** kindly give details of husband employment | Employee (Government)………………………01  Employee (Private sector)……………………..02  Self-employed (Farming/fishing)…….………..03  Self-employed (commerce)……………………04  Others, specify………………………………….. |  |
| **HB11.** what is your estimated household income  *Probe: income of both husband &wife* | Income per month …………………….  Income per year……………………….. | get both if possible |
| **HB12.** What do you have in your house? | \|  \| Yes \| No \| \| --- \| --- \| --- \| \| Electricity \|  \|  \| \| Radio \|  \|  \| \| Television \|  \|  \| \| Computer \|  \|  \| \| Refrigerator \|  \|  \| \| Gas cooker \|  \|  \| \| Toilet (KVIP) \|  \|  \| \| Toilet (WC) \|  \|  \| \| Electric generator \|  \|  \| \| None \|  \|  \| |  |
| **HB13.** Do you have a NHIS card and is it valid? | Yes, I have a valid NHIS card ………01  I have a NHIS card but it’s not valid …02  I don’t have a NHIS card ………….03 |  |

| birth history (ONLY FOR CHILDREN under 5 yrs) Bh |  |
| --- | --- |
| Now I would like to record the names of all of your births within the past five years, whether still alive or not.  *Record names of all of the births in BH1.Record twins and triplets on separate lines. (record name from the eldest to the youngest)*  bh | |

| BH  Line  No. | **BH1**.  What name was given to your (first/next) baby? | **BH2**.  Is (name) a boy or a girl?  1 Boy  2 Girl | **BH3**.  In what month and year was (name) born?  Probe: What is his/her birthday? | | **bh4**  Is (name) still alive?  1 Yes  2 No | **BH5**.  How old is (name) | **BH6**.  If dead:  How old was (name) when he/she died?  If “1 year”, probe:  How many months old was (name)?  Record days if less than 1 month; record months if less than 2 years; or years | | **BH7**  **what was the cause of death** |
| --- | --- | --- | --- | --- | --- | --- | --- | --- | --- |
| nO. | Name | B G | Month | Year | B G | Age | Unit | Number |  |
| 01 |  | 1 2 | ___ ___ | ___ ___ ___ ___ | 1 2 | ___ ___ | Days 1  Months 2  Years 3 | ___ ___ | malaria …….. 01  Diarrhea …….02  pneumonia ……03  DK …………….04  Others, specify …………… |
|  |  |  |  |  |  |  |  |  |  |
| 02 |  | 1 2 | ___ ___ | ___ ___ ___ ___ |  | ___ ___ | Days 1  Months 2  Years 3 | ___ ___ | malaria …….. 01  Diarrhea …….02  pneumonia ……03  DK …………….04 |
|  |  |  |  |  | 1 2 |  |  |  | Others, specify …………… |
| 03 |  |  | ___ ___ | ___ ___ ___ ___ |  | ___ ___ | Days 1  Months 2  Years 3 | ___ ___ | malaria …….. 01  Diarrhea …….02  pneumonia ……03  DK …………….04 |
|  |  | 1 2 |  |  | 1 2 |  |  |  | Others, specify …………… |
| 04 |  |  | ___ ___ | ___ ___ ___ ___ |  | ___ ___ | Days 1  Months 2  Years 3 | ___ ___ | malaria …….. 01  Diarrhea …….02  pneumonia ……03  DK …………….04 |
|  |  | 1 2 |  |  | 1 2 |  |  |  | Others, specify ……………… |
| 05 |  | 1 2 | ___ ___ | ___ ___ ___ ___ | 1 2 | ___ ___ | Days 1  Months 2  Years 3 | ___ ___ | malaria …….. 01  Diarrhea …….02  pneumonia ……03  DK …………….04  Others, specify …………… |

| ANTENATAL and delivery Ad | | | | |
| --- | --- | --- | --- | --- |
| This module is to be administered to all women with a live birth in the 5 years preceding the date of interview.  Record name of last-born child from BH here _____________________.  Use this child’s name in the following questions, where indicated. | | | | |
| **Ad1.** Were you advised to visit health facility for antenatal care when you were pregnant last time?  (The latest pregnancy) | Yes 1  No 2 | | 2⇨AD3 | |
| **AD2.** Who advised you to visit health facility for antenatal care during your pregnancy?  (Choose only one who gave her the initial advice) | Doctor 01  Midwife 02  Community health nurse/officer 03  Community health volunteer 04  Family/relatives 05  Neighbors 06  Other (specify) 07 | |  | |
| **AD3.** Where did you receive antenatal care during that pregnancy?  Probe to identify the type of source.  If unable to determine whether public or private, write the name of the place.    (Name of place) | **Public sector**  Regional hospital 01  District hospital 02  Health centre 03  CHPS Compound 04  **Private Medical sector**  Private hospital 05  Private clinic 06  Did not receive antenatal ………………….07  Other (*specify*) 08 | | 7 ⇨AD7 | |
| **AD4**. How many weeks or months pregnant were you when you first received antenatal care for this pregnancy?  *Record the answer as stated by respondent.* | Weeks 1 __  Months 2 __  Don’t Know……………………………99 | |  | |
| **AD5**. How many times did you receive antenatal care during this pregnancy?  Probe to identify the number of times antenatal care was received. If a range is given, record the minimum number of times antenatal care received. | Number of times __ __ | |  | |
| **AD6**. As part of your antenatal care during this pregnancy, were any of the following done at least once:  [A] Were you weighed?  [B] Was your blood pressure measured?  [C] Did you give a urine sample?  [D] Did you give a blood sample? | Yes No  Weight 1 2  Blood pressure 1 2  Urine sample 1 2  Blood sample 1 2 | |  | |
| **AD7**. Where did you give birth to (*name*)?  Probe to identify the type of source.  If unable to determine whether public or private, write the name of the place.    (Name of place) | **Home**  Respondent’s home 01  Other home 02  **Public sector**  Regional hospital………………………….03  District hospital 04  Health centre 05  CHPS Compound 06  **Private Medical sector**  Private hospital 07  Private clinic 08  Other (*specify*) | | Probe further for type of facility attended | |
| **AD8**. Who assisted with the delivery of (*name*)? | Doctor 01  Midwife 02  Community Health Nurse/officer 03  Traditional birth attendant 04  Community health volunteer 05  Family/relative 06  No one 07  Other (*specify*) 08 | |  | |
| **AD9.** Did you ever have fever during pregnancy? | Yes ………………………………………….1  No …………………………………………..2 | | 2 ⇨PN1 (next part) | |
| **AD10.** When you had fever during the pregnancy, DID YOU HAVE BLOOD TAKEN FROM YOUR FINGER FOR malariaTESTING? | Yes 1  No 2 | | 2⇨Pn1 (next part) | |
| **AD11**. If you were tested for malaria, where did you get tested?  Probe to identify the type of source.  If unable to determine whether public or private, write the name of the place.    (Name of place) | **Home**  CHV home visit 01  CHN/O home visit 02  **Public sector**  Regional hospital………………………..03  District hospital 04  Health centre 05  CHPS Compound 06  **Private Medical sector**  Private hospital 07  Private clinic 08  Drug store…………………………………09  **Other (*specify*)** | |  | |
| **AD12.** was your results confirmed to be malaria when you got tested? | Yes 1  No 2 | | 2⇨PN1 (next part) | |
| **AD13.** Did you get malaria treatment when you were diagnosed as malaria? | Yes 1  No 2 | |  | |
| Post-natal health checks pn | | | | |
| This module is to be administered to all women with a live birth in the 5 years preceding the date of interview.  Record name of last-born child from BH here _____________________.  Use this child’s name in the following questions, where indicated. | | | | |
| **PN1.**  Do you think that it is important to bring your baby for the first post-natal care after delivery? | | Very important 01  Important 02  Not important 03  Don’t Know 99 | |  |
| **PN2.** did you receive post-natal care within 6 weeks after delivery? | | Yes 01  No 02 | | 01=> go to PN4 |
| **PN3.** Why did you not receive post-natal care after delivery? | | Didn’t know if it was necessary …01  Could not get access to health facility/services... 02  Financial constraints 03  Family/Friend’s opinion 04  Husband/Partner’s opinion…………………….05  Other (specify) 06 | | Go to cp1 (the next section) after this question |
| **PN4.** How many times did you receive post-natal care within 6 weeks after delivery?  Probe to identify the number of times postnatal care was received. If a range is given, record the minimum number of times postnatal care received. | | Number of times __ __ | |  |
| **PN5.** When was the first post-natal care after delivery? | | Within 48 hours 01  2-7 days 02  8-21 days 03  22-28 days 04  29-42 days …………………………………….05 | |  |
| **PN6.** Did you take your baby with you for the first postnatal care? | | Yes 01  No 02 | |  |
| **PN7.** Who advised you to receive post-natal care within 48 hours after delivery?  **(**Choose only one who gave her the initial advice) | | Doctor 01  Midwife 02  Community health nurse/officer 03  Community health volunteer 04  Family/relative 05  No one 06  Other (specify) 07 | |  |
| **PN8.** Where did you receive the first post-natal care?  Probe to identify the type of source.  If unable to  determine whether public or private, write the name of the place.    (Name of place) | | **Home**  CHN/O home visit 01  **Public sector**  Regional hospital…………………………...02  District hospital 03  Health centre 04  CHPS Compound 05  **Private Medical sector**  Private hospital 06  Private clinic 07  **Other (*specify*)……………………………….08** | |  |

| contraception CP | | |
| --- | --- | --- |
| I would like to talk with you ABOUT FAMILY planning.  **CP1. Are** you pregnant now? | Yes 1  No 2  Unsure or DK 99 |  |
| **CP2.** COUPLES USE VARIOUS WAYS OR METHODS TO DELAY OR AVOID A PREGNANCY.  Have you heard of any of these methods? | Yes 1  No 2 | 2=>go to cp4 |
| **CP3.** which one have you heard of?  (Multiple responses possible) | Female sterilization 01  Male sterilization 02  IUD 03  Injectable 04  Implants 05  Pill 06  Male condom 07  Female condom 08  Standard days method 09  Rhythm method 10  Emergency Contraception……………………….11  Lactational Amenorrhea Method (LAM)……….12  Withdrawal………………………………………13  Other modern method (*specify*) 14  Other traditional method (specify) 15 |  |
| **CP4.** ARE YOU OR YOUR PARTNER CURRENTLY DOING SOMETHING OR USING ANY METHOD TO DELAY OR AVOID GETTING PREGNANT? | Yes 1  No 2 | 1=>GO TO CP7 |
| **CP5.** IF YOU ARE NOT USING ANY METHOD TO DELAY OR AVOID GETTING PREGNANT, HAVE YOU USED ANY METHOD IN THE PAST? | Yes 1  No 2 | 2=>GO TO CP11 |
| **CP6.** WHY DID YOU STOP USING THE METHODS? | 1) Wanted to get pregnant  2) Partner disagreed to use it  3) Didn’t feel comfortable using it any more  4) Costly  5) Side effects  6) Religious Reasons  7) Cultural Reasons  8) It was not effective  9) Others |  |
| THERE ARE DIFFERENT METHODS.  PLEASE CHECK ACCORDING TO YOUR EXPERIENCE.  **CP7.** WHICH ONE HAVE YOU USED BEFORE?  **CP8.** WHICH ONE ARE YOU USING NOW?  [A] FEMALE STERILIZATION  [B] MALE STERILIZATION  [C] IUD  [D] INJECTABLE  [E] IMPLANTS  [F] PILLS  [G] MALE CONDOM  [H] FEMALE CONDOM  [I] STANDARD DAYS METHOD  [J] RHYTHM METHOD  [K] emergency Contraception  [L] Lactational Amenorrhea Method (LAM)  [m] Withdrawal method  [n] OTHER MODERN METHODS  [o] OTHER TRDITIONAL METHOD | Yes 1  No 2   \| No. \| CP7 \| CP8 \| \| --- \| --- \| --- \| \| A \|  \|  \| \| B \|  \|  \| \| C \|  \|  \| \| D \|  \|  \| \| E \|  \|  \| \| F \|  \|  \| \| G \|  \|  \| \| H \|  \|  \| \| I \|  \|  \| \| J \|  \|  \| \| K \|  \|  \| \| L \|  \|  \| \| M \|  \|  \| \| N \|  \|  \| \| O \|  \|  \| |  |
| **CP9.** If you are using contraception now what motivated you to use it | 1) My own choice  2) Family’s recommendation  3) Health worker’s recommendation  4) CHV’s recommendation  5) Friend/neighbor’s recommendation  6) Husband/Partner’s recommendation  7) Am not using it now  8) Others |  |
| **CP10.** WHERE DID YOU RECEIVE OR PURCHASE the contraception method you are using or used  (Multiple responses possible) | Home  CHV home visit 01  CHN home visit 02  Public sector  Regional hospital…………………………..03  District hospital 04  Health centre 05  CHPS Compound 06  Private Medical sector  Private hospital 07  Private clinic 08  Drug store………………………………09  Other (*specify*) 10 |  |
| **CP11.** DO YOU WANT TO USE IT IN THE FUTURE? | Yes 1  No 2 |  |

[A] Female Sterilization: Women can have an operation to avoid having any more children.

[B] Male Sterilization: Man can have an operation to avoid having any more children.

[C] IUD: Women can have a loop or coil placed inside them by a doctor or a nurse which can prevent pregnancy for one or more years.

[D] Injectable: Women can have an injection by a health provider that stops them from becoming pregnant for one or more months

[E] Implants: Women can have one or more small rods placed in their upper arm by a doctor or nurse which can prevent pregnancy for one or more years

[F] Pill: Women can take a pill every day to avoid becoming pregnant.

[G] Male Condom: Men can put a rubber on their penis before sexual intercourse.

[H] Female condom: Women can place a sheath in their vagina before sexual intercourse.

[I] Other modern methods;

Emergency Contraception: As an emergency measure, within three days after they have unprotected sexual intercourse, women can take special pills to prevent pregnancy.

Standard Days Methods: A women uses a string of colored beads to know the days she can get pregnant. On the days she can get pregnant, she uses a condom or does not have sexual intercourse.

[J] Any traditional method;

Lactational Amenorrhea Method (LAM): Up to six months after childbirth, before the menstrual period has return, women use a method requiring frequent [L] breastfeeding day and night.

Rhythm Method: To avoid pregnancy, women do not have sexual intercourse on the days of the month they think they can get pregnant.

Withdraw: Men can be careful and pull out before climax.

| child healtH CH | | | | | |
| --- | --- | --- | --- | --- | --- |
| This module is to be administered to all women with a live birth in the 5 years preceding the date of interview. Record name of last-born child from BH here _____________________.  Use this child’s name in the following questions, where indicated. | | | | | |
| For vaccinations, request to show (name)’s vaccination card politely | | | | | |
| **CH1.** has (name) shown any of these symptoms in the past six (6) months?  (Multiple responses possible) | | Not able to drink or breastfeed……...01  Develops a fever…………………….02  Breathing problem (difficult or fast)….03  Watery or loose stool………………….04  Blood in stool…………………………05  Others (specify) ……………………….06  None…………………………………...07 | 07=>go to ch4 | | |
| **CH2 did you seek for** treatment when (name) showed those symptoms? | | Yes…………………………………1  No………………………………….2 | 2=>CH4 | | |
| **CH**3. where did you first seek treatment when (name) was sick? | | **Home**  CHV home visit………………01  CHN home visit…02  **Public sector**  Regional hospital…………………..03  District hospital…………………....04  Health centre………………………05  CHPS Compound………………….06  **Private Medical sector**  Private hospital……...……………..07  Private clinic…………………...…..08  Drug store…...……………………..09  Other (*specify*)………………….. |  | | |
| **CH4.** What is the main source of drinking for (name)? | | Public stand pipe…………….01  Boreholes……………………..02  Hand- dug well………………..03  Stream/River………………….04  Water tanker………………….05  Sachet Water………………….06  Bottled Water………………….07  Others (specify) ………………….08 |  | | |
| **CH5.** Can you provide the weighing card of (name) to me now | | Yes…………………………….01  No …………………………....02 | 2=>DA1 | | |
| **CH6.** What is the most recently measured weight of (name)? | | ……….kg |  | | |
| **CH7.** what was the date (name)’s weight was measured | |  |  | | |
| **DIARRHEA (in under-5 children) DA** | | | | |  |
| This module is to be administered to all women with a live birth in the 5 years preceding the date of interview. Record name of last-born child from BH here _____________________.  Use this child’s name in the following questions, where indicated. | | | | |  |
| **DA1.** Has *(name)* had 3 or more watery or loose stools per day lasting for 3 or more days in the past 2 week? | Yes 1  No 2 | | | 2=> ma1 (next section) |  |
| **DA2.** Have you received ors or any recommended homemade fluids for (name)?  *****Recommended homemade fluids:  coconut water, rice water or mashed kenkey | Yes 1  No 2 | | | 2=> Go to ma1(next section) |  |
| **DA3.** Which solution did you take for (name)? | Oral rehydration solution 01  Coconut Water 02  Rice Water 03  Mashed Kenkey 04  Corn related fluids…………………………...05  Other Specify ………………………………… | | | 2,3,4,5=>go to da5 |  |
| **DA4**. Where did you get the ors from?  Probe to identify the type of source.  If unable to determine whether public or private, write the name of the place.    (Name of place) | **Home**  CHV home visit 01  CHN home visit 02  **Public sector**  Regional hospital…………………………..03  District hospital 04  Health centre 05  CHPS Compound 06  **Private Medical sector**  Private hospital 07  Private clinic 08  Drug store…………………………………09  **Other (*specify*)** | | | Go to DA6 after da4 |  |
| **DA5.**  Why did you not take ors but homemade fluids? | ORS was expensive 01  Difficulty in accessing ORS 02  Homemade fluids have better efficacy 03  Didn’t know about ORS 04  Other (specify) 05 | | |  |  |
| **DA6.** Have you sought further treatment after giving ors or fluids to (name)? | Yes 1  No 2 | | |  |  |

| **MALARIA (in under-5 children) MA** | | |
| --- | --- | --- |
| This module is to be administered to all women with a live birth in the 5 years preceding the date of interview. Record name of last-born child from BH here _____________________.  Use this child’s name in the following questions, where indicated. | | |
| **MA1**. In the last 2 weeks, has (name) been ill with a fever at any time? | Yes 1  No 2 | 2=> CV1(next section) |
| **MA2**. AT ANY TIME DURING THE ILLNESS, DID (NAME) HAVE BLOOD TAKEN FROM HIS/HER FINGER OR HEEL FOR TESTING? | Yes, for Malaria 1  Yes for other reasons 2  No……………………………………………..3 | 2,3=> go to MA4 |
| **MA3**. If (name) was tested for malaria, where did (name) get tested?  Probe to identify the type of source.  If unable to determine whether public or private, write the name of the place.    (Name of place) | **Home**  CHV home Visit 01  CHN home Visit 02  **Public sector**  Regional hospital…………………………..03  District hospital 04  Health centre 05  CHPS Compound 06  **Private Medical sector**  Private hospital 07  Private clinic 08  Drug store…………………………………09  **Other (*specify*)** |  |
| **MA4.** did you seek treatment for the illness from any source? | Yes 1  No 2 | 2=> go to CV1(next section) |
| **MA5**. Where did you seek medical treatment for *(name)* when*(name)* HAD malaria?  Probe to identify the type of source.  If unable to determine whether public or private, write the name of the place.    (Name of place) | **Home**  Used left over syrups and pills at home…..01  Prepare herbal Concoction at home………02  Traditional Healers……………………….03  **Public sector**  Regional hospital…………………………04  District hospital 05  Health centre 06  CHPS Compound 07  **Private Medical sector**  Private hospital 08  Private clinic 09  Drug store………………………………...10  **Other (*specify*)** |  |

| coMMUNITY HEALTH VOLUNTEERS CV | | |
| --- | --- | --- |
| **CV1**.Do you have community health volunteers (CHV) working in this community? | Yes 1  No 2  Don’t Know 99 |  |
| **CV2.** I would like to talk with you about the existence and activities of community health volunteers.  What do you think are the chv’s role in the community?  (Multiple choice possible) | Assisting CHN/CHO 01  Health promotion/education 02  Disease surveillance 03  Registration of pregnant women 04  Anti Natal Care 06  Post Natal Care 07  Delivery 08  Disease prevention activity in the community 09  Medicine prescription 10  Diagnosis of minor illness 11  Diagnosis of severe illness 12  Birth and death registration…………………..13  Other Specify ………………………………… |  |
| **CV3**. when was the last time you were visited by a chv? | Within 1 months…………………………….01  1-2 Months………………………………….02  2-3 months………………………………….03  3-6 months………………………………...04  Over 6 months………………………………05  Never visited by a CHV……………………06 | 06=>CV8 |
| **CV4.** How many times did a chv visit your house in the last 6 months? | …………. |  |
| **CV5.** What was the gender of CHV who visited your house? | Female 1  Male 2 |  |
| **CV6.**  how long did the CHV stay during last home visit? | …………. Hour & ……….. Minute |  |
| **CV7.** Did a chv take records during the last home visit | Yes 1  No………………………………………………2 |  |
| **CV8**. has a chv provided you any health promotion or preventive education? | Yes 1  No 2 | 2=>CV10 |
| **CV9.**  What kind?  (multiple choice possible) | Family planning & contraception 1  Anti Natal Care 2  Post Natal Care 3  Malaria prevention 4  Diarrhea prevention 5  Diarrhea treatment……………………………...6  WASH 7  Nutrition 8  Child Health 9 |  |
| **CV10.**  Have you received any health product from CHV? | Yes 1  No 2 | 2=>CV12 |
| **CV11.** What have you received from CHV?  **(**Multiple choice possible) | Mosquito net 1  ORS 2  Aqua tabs……………………………………….3  Other (specify) |  |
| **CV12**. in your opinion What was the most impressive community health activity done by chv? | Home visit………………………….................01  Supporting CHN outreach……………….……02  Community mobilization for community activity……………………………………......03  Distribution of health products ………………04  None…………………………………………..05  Other (specify)…………………………… |  |
| **CV13**. Do you think CHV helps keep you healthy? | Yes 01  No 02  Have not had interactions with CHV…………03 |  |
| **CV14.** Do YOU think chv contributes to promote health in the community? | Yes 01  No 02  Have not had interactions with CHV…………03 |  |
| **CV15.** Overall how will you rate the services delivered by the chv to you and your family? | Very Satisfactory…………………………...01  Satisfactory…………………………………02  Fairly Satisfactory…………………………..03  Not Satisfactory…………………………… 04  Poor…………………………………………05  Have not have interactions with CHV………05 |  |

| **Interviewer’s observation**  (the interviewer must check the respond fidelity of the mother.) |
| --- |

Record name of _____________________.

| *Record the time taken for interview*. | Hour and minutes __ __ : __ __ |  |
| --- | --- | --- |
