# Supplementary material for: Evaluating the impact of community health volunteer home visits on child diarrhea and fever in the Volta Region, Ghana: A cluster-randomized controlled trial
Source: PLoS Med. 2019 Jun 14;16(6):e1002830. doi: 10.1371/journal.pmed.1002830 (PMC6568387; doi:10.1371/journal.pmed.1002830)
Supplement: S1 Table — (DOCX) [file pmed.1002830.s004.docx]

**S1 Table. Longitudinal Generalized Estimating Equation (GEE) analysis of the effect of the community health volunteer intervention on diarrhea, fever, malaria testing for fever, oral rehydration salts (ORS) treatment for diarrhea, and family planning prevalence at 6 and 12 months of follow-up**

|  | Relative risk* at  6 and 12 months  (95% CI) | p-value | Adjusted Relative risk† at  6 and 12 months (95% CI) | p-value |
| --- | --- | --- | --- | --- |
|  |  |  |  |  |
| ***Primary Outcomes*** | | | | |
| 14-day Diarrhea prevalence | 0.77 (0.51-1.16) | 0.21 | 0.79 (0.52-1.21) | 0.28 |
| 14-day Fever prevalence | 0.87 (0.64-1.18) | 0.38 | 0.91 (0.68-1.23) | 0.55 |
| ***Secondary outcomes*** | | | | |
| ORS for diarrhea cases | 0.92 (0.77-1.11) | 0.41 | 1.04 (0.83-1.29) | 0.76 |
| Malaria test for fever cases | 1.06 (0.78-1.44) | 0.70 | 1.15 (0.81-1.64) | 0.42 |
| Family planning | 1.00 (0.86-1.15) | 0.52 | 0.99 (0.88-1.12) | 0.87 |

* Accounting for cluster and stratified randomization

† Diarrhea: adjusted for baseline diarrhea, and wealth, caregiver's education, child sex, age, water source, latrine, clustering effect, and stratification; Fever: adjusted for baseline fever, and wealth, caregiver's education, child sex, age, clustering effect, and stratification; Malaria test and ORS treatment: adjusted for baseline value, clustering effect, and stratification; Family planning practices: adjusted for baseline values, mothers' education level, clustering effect, and stratification.
