# Supplementary material for: Evaluating the impact of community health volunteer home visits on child diarrhea and fever in the Volta Region, Ghana: A cluster-randomized controlled trial
Source: PLoS Med. 2019 Jun 14;16(6):e1002830. doi: 10.1371/journal.pmed.1002830 (PMC6568387; doi:10.1371/journal.pmed.1002830)
Supplement: S2 Table — (DOCX) [file pmed.1002830.s005.docx]

**S2 Table. Recall of the 10 key messages of CHV program at 6 and 12 months follow-up**

|  | **6-month Follow-up** | **12-month Follow-up** | Mean difference from  6 to 12 months | p-value |
| --- | --- | --- | --- | --- |
|  | % (n/N) or Mean (SD) | % (n/N) or Mean (SD) |  |  |
| ***Key message recalled*** |  |  |  |  |
| Family planning | 47.5% (235/495) | 33.4% (201/601) | -14.0% (-19.8%, -8.2%) | <0.001 |
| Antenatal care | 38.4% (190/495) | 16.1% (97/601) | -22.2% (-27.4%, -17.0%) | <0.001 |
| Postnatal care | 44.8% (222/495) | 19.8% (119/601) | -25.0% (-30.5%, -19.6%) | <0.001 |
| Malaria prevention | 75.4% (373/495) | 66.4% (399/601) | -9.0% (-14.3%, -3.6%) | 0.001 |
| Diarrhea prevention | 70.7% (350/495) | 66.7% (401/601) | -4.0% (-9.5%, 1.5%) | 0.16 |
| Diarrhea management | 47.5% (235/495) | 25.1% (151/601) | -22.3% (-28.0%, -16.7%) | <0.001 |
| Exclusive breastfeeding | 80.6% (399/495) | 76.5% (460/601) | -4.1% (-8.9%, 0.8%) | 0.10 |
| Anaemia prevention | 79.8% (395/495) | 59.1% (355/601) | -20.7% (-26.0%, -15.4%) | <0.001 |
| Skilled delivery | 8.1% (40/465) | 2.5% (15/601) | -5.6% (-8.3%, -2.9%) | <0.001 |
| Malaria management | 32.1% (159/495) | 12.8% (77/601) | -19.3% (-24.2%, -14.4%) | <0.001 |
| ***Mean number of key messages recalled (SD)*** | 5.3 (2.6) | 3.8 (1.9) | -1.46 (-1.73, -1.20) | <0.001 |
