# Supplementary material for: Evaluating the impact of community health volunteer home visits on child diarrhea and fever in the Volta Region, Ghana: A cluster-randomized controlled trial
Source: PLoS Med. 2019 Jun 14;16(6):e1002830. doi: 10.1371/journal.pmed.1002830 (PMC6568387; doi:10.1371/journal.pmed.1002830)
Supplement: S3 Table — (DOCX) [file pmed.1002830.s006.docx]

S3 Table. Ante-natal and pre-natal care, and case management of malaria of pregnant women

|  | **Baseline** | | **6-month follow-up** | | **12-month follow-up** | |
| --- | --- | --- | --- | --- | --- | --- |
|  | Intervention (N=999) | Control (N=957) | Intervention (N=854) | Control (N=806) | Intervention (N=825) | Control (N=784) |
| Percentage of pregnant women taking ante-natal care of pregnant women (4 or more times)* | 77% (774/999) | 79% (752/957) | 70% (74/106) | 70% (58/83) | 76% (39/51) | 84% (49/58) |
| Percentage of women who had post-natal care within 6 weeks after delivery* | 91% (905/999) | 91% (868/957) | 85% (90/106) | 81% (67/83) | 78% (40/51) | 86% (50/58) |
| Percentage of women who had the first post-natal care within 48 hours after delivery* | 31% (312/999) | 35% (339/957) | 42% (44/106) | 52% (43/83) | 59% (30/51) | 57% (33/58) |
| Percentage of pregnant women with fever taking rapid diagnostic test for malaria^†^ | 73% (386/531) | 75% (386/517) | 76% (47/62) | 80% (39/49) | 94% (16/17) | 91% (29/32) |

* Denominator: the number of women who experienced a delivery between surveys. For the baseline, the denominator is identical to the number of each arm.

† Denominator: the number of women who had fever during their pregnancy between surveys. For the baseline, the denominator is the number of women who had fever during their latest pregnancy.
