# Supplementary material for: Evaluating the impact of community health volunteer home visits on child diarrhea and fever in the Volta Region, Ghana: A cluster-randomized controlled trial
Source: PLoS Med. 2019 Jun 14;16(6):e1002830. doi: 10.1371/journal.pmed.1002830 (PMC6568387; doi:10.1371/journal.pmed.1002830)
Supplement: S1 Text — (DOCX) [file pmed.1002830.s007.docx]

**S1 Text. Community Health Volunteer Program Outline**

***Selection criteria for community health volunteers (CHVs)***

- Eligibility: CHVs should be residents of the community.
- CHVs who met the following criteria were recommended and endorsed by the community leader or committee:

(1) S/he could read and write.

(2) S/he had a spirit of volunteerism.

(3) S/he had previous experience as a health volunteer (preferable).

***Training***

- All CHVs were provided with 5 days of training, including 2 days of field-based practice.
- CHVs with a poor score on the post-training test were given additional training for 1 day and self-paced learning with course materials was suggested.
- CHVs were offered refresher training at review workshops every 6 months, in addition to monthly refreshers at each Community-based Health Planning and Services (CHPS) compound.

***Supervision and monitoring***

- Facilitative supervision was monthly provided by the monitoring team, made up of the District Health Management Team (DHMT) and project team members.
- Community health nurses and officers (CHNs/CHOs) at each CHPS compound supervised CHVs who worked within its catchment area. (The CHN/O to CHV ratio varied from 1:2 to 1:10.)
- CHNs and the monitoring team occasionally did a spot check on CHVs’ home visit practices.
- Key stakeholders (community leaders, CHNs/CHOs, DHMT staff members, and sub-district health staff members) were invited to quarterly review workshops for follow-up on the progress. All CHVs joined the workshop every 6 months for peer review and refresher training.

***Core tasks***

- Conduct home visits to deliver health education with 10 key messages (at least once per 2 months per household; each visit was expected to last for a minimum of 30 minutes)
- Key Messages included:

| 1. Mothers must wash their hands with soap and running water at 5 critical moments: before cooking, before eating, before child feeding, after defecating, and after handshaking; Proper handwashing prevents diarrhea; Avoid open defecation and keep latrines clean. 2. Use oral rehydration salts (ORS) and Zinc tablets; If ORS is not available, use rice water, coconut water or mashed *kenkey.* 3. Every person, especially pregnant woman and child under-5 should sleep under insecticide-treated net (ITN); Pregnant women should take intermittent preventive treatment (IPT) to prevent malaria. 4. People diagnosed positive for malaria should go to a health facility for treatment. 5. Family planning prevents unwanted pregnancy; Go to a health facility to find the best method for you. 6. Go to a health facility for antenatal care services when you get pregnant. 7. Delivery at health facility keeps you and your baby safe and healthy. 8. Go to a health facility with your baby for postnatal care services in the first week after birth. 9. To keep your baby healthy, practice exclusive breastfeeding for 6 months. 10. Eat green vegetables and fruits; Go to a health facility to take iron tablets; Deworm your children every three months; Participate in community outreach program. |
| --- |

- Offer counseling to the household members based on their needs
- Deliver oral rehydration salts (ORS) packets and zinc tablets to children with diarrhea as a first-aid treatment, then refer them to CHPS compounds or the closest health facilities
- Conduct a blood test with a rapid diagnostic test kit for malaria in those with fever, then refer them to CHPS compounds or the closest health facilities for treatment if the test result was positive
- Refer cases that required treatment or special management to CHNs/CHOs
- Mobilize community members to participate in community-wide health activities including child welfare clinics (CWCs) and National Sanitation Day events
- Support CHNs/CHOs at CWCs for immunization, micronutrient supplementation, anthropometric measurements, and nutrition and hygiene education for under-five children and their mothers

***Catchment***

- Each CHV covered 40 households.

***Incentives***

Monthly, 10 USD worth of airtime and food items was provided for each CHV. The food items cost 8.7 USD (30 GHC) and airtime 1.5 USD (5 GHC). *(The exchange rate on August 1, 2015 was applied: 1 USD = 3.46 GHC.)*

No monetary incentive was provided.

***Results of CHVs’ activation***

- Criteria and results for assessing the quality of CHVs’ activation:

1. Tests before/after the initial training: CHVs who scored 75% or more were considered qualified.

Proportion of qualified CHVs before activation: 8% (pre-training test); 47% (post-training test); 69% (follow-up test); 95% (second follow-up test)

1. Monthly review meeting attendance rate of CHVs: 94% (average for 12 months)
2. Proportion of CHVs with constant recording of logbooks: 94%, 94%, 93%, and 91% at 3, 6, 9, and 12 months, respectively
3. CHVs’ self-reported coverage of home visits: 74%, 69%, 69%, and 66% at 3, 6, 9, and 12 months, respectively (dose delivered)
4. CHVs’ participation in CWCs: 68% (average for 12 months)
5. Retention rate of CHVs**:** 100%, 92%, 91%, 87% at 3, 6, 9, and 12 months, respectively

***Demographic profiles of CHVs***

- Female: 26% (20/77)
- Age 20-39: 64% (50/77)
- Married: 75% (58/77)
- Education:

No education or graduated primary school: 12% (9/77)

Graduated junior high school: 52% (40/77)

Graduated senior high school or with higher education: 36% (28/77)

- Occupation:

Farmers: 27% (22/82*)

Small-scale petty traders or self-employed: 51% (42/82*)

**Some CHVs reported having more than one occupation.*

- Religion:

Christianity: 83% (64/77)

Traditional: 12% (9/77)

- Previous volunteering experience: 47% (36/77)
